# Innate immunity of vascular smooth muscle cells contributes to two-wave inflammation in atherosclerosis, twin-peak inflammation in aortic aneurysms and trans-differentiation potential into 25 cell types

**DOI:** 10.3389/fimmu.2023.1348238

**Published:** 2024-01-24

**Authors:** Qiaoxi Yang, Fatma Saaoud, Yifan Lu, Yujiang Pu, Keman Xu, Ying Shao, Xiaohua Jiang, Sheng Wu, Ling Yang, Ying Tian, Xiaolei Liu, Avrum Gillespie, Jin Jun Luo, Xinghua Mindy Shi, Huaqing Zhao, Laisel Martinez, Roberto Vazquez-Padron, Hong Wang, Xiaofeng Yang

**Affiliations:** ^1^ Lemole Center for Integrated Lymphatics and Vascular Research, Department of Cardiovascular Sciences, Lewis Katz School of Medicine at Temple University, Philadelphia, PA, United States; ^2^ Beloit College, Beloit, WI, United States; ^3^ College of Letters & Science, University of Wisconsin-Madison, Madison, WI, United States; ^4^ Center for Metabolic Disease Research and Thrombosis Research, Department of Cardiovascular Sciences, Lewis Katz School of Medicine at Temple University, Philadelphia, PA, United States; ^5^ Department of Medical Genetics and Molecular Biochemistry, Lewis Katz School of Medicine at Temple University, Philadelphia, PA, United States; ^6^ Section of Nephrology, Hypertension, and Kidney Transplantation, Department of Medicine, Lewis Katz School of Medicine at Temple University, Philadelphia, PA, United States; ^7^ Department of Neurology, Lewis Katz School of Medicine at Temple University, Philadelphia, PA, United States; ^8^ Department of Computer and Information Sciences, College of Science and Technology at Temple University, Philadelphia, PA, United States; ^9^ Center for Biostatistics and Epidemiology, Department of Biomedical Education and Data Science, Lewis Katz School of Medicine at Temple University, Philadelphia, PA, United States; ^10^ DeWitt Daughtry Family Department of Surgery, Leonard M. Miller School of Medicine, University of Miami, Miami, FL, United States

**Keywords:** VSMCs, vascular inflammation, trained immunity, nuclear stress, trans-differentiation

## Abstract

**Introduction:**

Vascular smooth muscle cells (VSMCs) are the predominant cell type in the medial layer of the aorta, which plays a critical role in aortic diseases. Innate immunity is the main driving force for cardiovascular diseases.

**Methods:**

To determine the roles of innate immunity in VSMC and aortic pathologies, we performed transcriptome analyses on aortas from ApoE^–/–^ angiotensin II (Ang II)-induced aortic aneurysm (AAA) time course, and ApoE^–/–^ atherosclerosis time course, as well as VSMCs stimulated with danger-associated molecular patterns (DAMPs).

**Results:**

We made significant findings: *1)* 95% and 45% of the upregulated innate immune pathways (UIIPs, based on data of 1226 innate immune genes) in ApoE^–/–^ Ang II-induced AAA at 7 days were different from that of 14 and 28 days, respectively; and AAA showed twin peaks of UIIPs with a major peak at 7 days and a minor peak at 28 days; *2)* all the UIIPs in ApoE^–/–^ atherosclerosis at 6 weeks were different from that of 32 and 78 weeks (two waves); *3)* analyses of additional 12 lists of innate immune-related genes with 1325 cytokine and chemokine genes, 2022 plasma membrane protein genes, 373 clusters of differentiation (CD) marker genes, 280 nuclear membrane protein genes, 1425 nucleoli protein genes, 6750 nucleoplasm protein genes, 1496 transcription factors (TFs) including 15 pioneer TFs, 164 histone modification enzymes, 102 oxidative cell death genes, 68 necrotic cell death genes, and 47 efferocytosis genes confirmed two-wave inflammation in atherosclerosis and twin-peak inflammation in AAA; *4)* DAMPs-stimulated VSMCs were innate immune cells as judged by the upregulation of innate immune genes and genes from 12 additional lists; *5)* DAMPs-stimulated VSMCs increased trans-differentiation potential by upregulating not only some of 82 markers of 7 VSMC-plastic cell types, including fibroblast, osteogenic, myofibroblast, macrophage, adipocyte, foam cell, and mesenchymal cell, but also 18 new cell types (out of 79 human cell types with 8065 cell markers); *6)* analysis of gene deficient transcriptomes indicated that the antioxidant transcription factor NRF2 suppresses, however, the other five inflammatory transcription factors and master regulators, including AHR, NF-KB, NOX (ROS enzyme), PERK, and SET7 promote the upregulation of twelve lists of innate immune genes in atherosclerosis, AAA, and DAMP-stimulated VSMCs; and *7)* both SET7 and trained tolerance-promoting metabolite itaconate contributed to twin-peak upregulation of cytokines in AAA.

**Discussion:**

Our findings have provided novel insights on the roles of innate immune responses and nuclear stresses in the development of AAA, atherosclerosis, and VSMC immunology and provided novel therapeutic targets for treating those significant cardiovascular and cerebrovascular diseases.

## Introduction

Atherosclerosis is a chronic inflammatory and autoimmune disease of the arterial wall, which is one of the primary causes underlying pathologies for cardiovascular and cerebrovascular diseases such as myocardial infarction (MI), stroke, and peripheral arterial disease (1, 2). About 695,000 people in the United States died from cardiovascular disease (CVD) in 2021, or 20% of all deaths in the country, as the CDC reported (https://www.cdc.gov/heartdisease/facts.htm). Significant progress has been made in understanding the pathogenesis of atherosclerosis. We and others reported that innate and adaptive immune responses play essential roles in promoting atherogenic progression (3–7), including endothelial cell activation (8, 9) and trans-differentiation into innate immune cells (7, 10, 11), vascular smooth muscle cell (VSMCs) phenotype switching (12, 13), proinflammatory monocyte differentiation (14, 15), macrophage polarization (16, 17), CD4^+^Foxp3^+^ regulatory T cell (Treg) death (18–20), Treg plasticity (21), Treg immunosuppression sustainability (6), trained immunity (4, 7), secretomes (22–25), and metabolomic analyses (26). However, an important question remains: whether innate immune responses in the early and later stages of atherosclerosis have any stage-specificities.

Similarly, abdominal and thoracic aortic aneurysms are the second most common disease affecting the aorta after atherosclerosis, the fifth leading cause of death in individuals aged ≥ 55 years, and the 19th leading cause of death overall (27, 28), according to CDC Statistics (https://www.cdc.gov/injury/wisqars/LeadingCauses.html). Aortic aneurysms are defined as a ≥ 50% localized increase in the observed diameter of the aorta compared with the same aortic segment in age- and sex-matched healthy individuals (a ratio of the observed to the expected diameter of ≥ 1.5). Abdominal aortic aneurysms (AAA) are much more common than thoracic aortic aneurysms (TAA) (29, 30). Pathological findings indicated that AAAs have the features of vascular remodeling and the loss of VSMCs (30). Despite many progresses in determining the roles of genetic risk factors (31), inflammatory stimuli, inflammasome activation and pyroptosis (32, 33), monocytes and macrophages (17, 34), Tregs (35), cell death (36), reactive oxygen species (ROS) (37–39), matrix metalloproteinases (MMPs), microRNAs, circular RNAs (40) and epigenetics (38), VSMC phenotypic switching (13) and calcification (41), advanced technologies for gene discovery (31), and animal models (42, 43), a significant question remains: which innate immune mechanisms are underlying the inflammatory signal amplification for the localized dilatations that happened in specific areas of the aorta (42).

Vascular smooth muscle cells (VSMCs) are a major cell type present at all stages of an atherosclerotic plaque. Contractile VSMCs recruited from the media undergo phenotypic conversion to proliferative synthetic cells that generate extracellular matrix to form the fibrous cap and hence stabilize plaques (44). We previously reported that neointima hyperplasia in carotid artery, mainly mediated by VSMCs, is decreased significantly in 5/6 nephrectomy established chronic kidney disease (CKD) model in inflammatory caspase-1 knockout mice compared to CKD wild-type controls (12); contractile protein markers are decreased by 50 – 80% in human VSMCs stimulated by uremic serum from CKD patients (13, 45); and secretomic genes (23), including proinflammatory caspase-1-gasdermin D (GSDMD) secretome and caspase-4/11-GSDMD secretome are upregulated in angiotensin II (Ang II)-treated VSMCs in addition to secretome upregulation in aortic diseases and vascular endothelial cells infected by Middle-East respiratory syndrome coronavirus (MERS-CoV) (22). Others also reported that VSMCs in CVDs can be trans-differentiated into other seven cell types, including mesenchymal stem cell-like cells, fibroblasts-like cells, myofibroblast-like cells, macrophage-like cells, foam cell-like cells, osteogenic-like cells, and adipocyte-like cells (46–48). Previously, we identified vascular endothelial cells (ECs) as innate immune cells by playing 11 macrophage-played innate immune functions (3, 49) and upregulating innate immune genes in ECs (3, 10, 11, 49, 50). We also reported innate immune sensor caspase-1 and inflammasome functions in human aortic ECs (HAECs) related to atherosclerosis (8, 33) and innate immune memory function (also termed trained immunity) (4, 5) in HAECs in response to the stimulation of gut microbiota-generated uremic toxin trimethylamine N-oxide (TMAO) (7, 45, 51). However, a few important questions remain unknown: i) whether VSMCs are innate immune cells in aortic diseases; ii) whether innate immune genes are upregulated in VSMCs responding to danger-associated molecular patterns (DAMPs) (2) stimulation; and iii) whether nuclear localized protein genes (nuclear membrane, nucleoli, and nucleoplasm) are upregulated in aortic diseases and VSMCs stimulated by DAMPs.

The nucleus is the most important cellular organelle (52), which plays a vital role in all the pathophysiological processes (53), including genome stability maintenance, DNA replication and repair (54), RNA transcription, RNA processing and splicing (55, 56), epigenetic histone modifications (53), and cell death responses (36, 57). We previously reported a list of papers and reviews on nuclear stresses: *a)* innate immune sensor proinflammatory caspase-1 migrates from the cytosol into the nucleus (58) and become activated in HAECs in response to the stimulation of conditional DAMP (59, 60) lysophosphatidylcholine (LysoPC) and its associated nuclear stresses (53, 58); *b)* the majority of histone modification enzymes are downregulated in metabolic diseases (61); c) many caspase-1 substrates, caspase-1 interaction proteins, and inflammasome components are localized in the nucleus (58); *d)* the majority of nuclear receptor family members with transcription factor function and environment stimuli-sensing functions are localized in the nucleus (62); *e)* nuclear metabolic stresses in the nucleus and other organelles can be sensed by ROS systems (37); and *f)* DNA damage and DNA repair regulators are significantly modulated in metabolic CVDs (54). It has been reported that nucleoli are a nuclear stress response membraneless organelle that forms post-translationally (63–66). However, global nuclear stresses modulated transcriptionally in CVDs and VSMCs remain poorly characterized. In addition, the detailed molecular relationships between innate immune responses and transcriptomic changes of three lists of nuclear proteins, including nuclear membrane proteins, nucleoli proteins, and nucleoplasm proteins, in the pathologies of aortic diseases and the innate immune responses of VSMCs to various DAMPs have never been examined.

**Figure 1:**
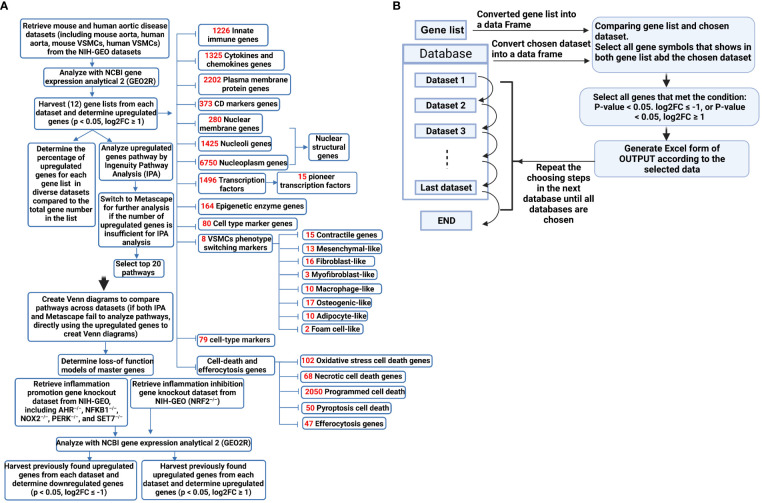
**(A)** A flow chart for transcriptomic data analyses in unbiased (Ingenuity Pathway Analysis, IPA, or Metascape for groups of smaller upregulated genes) and knowledge-based (focused) approaches. **(B)** Using Python to process data improved work efficiency and prevented potential errors in manual processing. The data processing involved using the pandas library (https://pandas.pydata.org/) in Python to convert the data into DataFrame format. The genes were filtered based on specified conditions (e.g. p-value < 0.05, log2FC ≥ 1), and were subsequently organized as the screened data into separate Excel files for subsequent statistical analysis.

Innate immune cells play significant roles in promoting the pathogenesis of atherosclerosis (2, 33, 49, 67), aortic aneurysms (22, 30, 34), and other vascular inflammation (68). However, the roles of transcriptomic remodeling of innate immune genes in aortic diseases and VSMC responses to DAMPs remain poorly characterized. To address the above-mentioned key knowledge gaps, we collected nearly 25 transcriptomic datasets, including the apolipoprotein E deficient (ApoE^–/–^) angiotensin II (Ang II)-induced abdominal aortic aneurysm (AAA) mouse model (10, 69), the ApoE^–/–^ atherosclerotic aorta in high fat diet (HFD) feeding for three time points, including 6 weeks, 32 weeks, and 78 weeks, the ApoE^–/–^ Ang II-induced AAA examined on three time points, including 7 days, 14 days, and 28 days, and VSMCs stimulated by various DAMPs. As shown in **Figure 1**, we performed extensive transcriptomic analyses to determine the roles of innate immune genes, nucleus-localized protein genes, and a list of master gene-deficient (^–/–^) datasets. In addition, we screened the transcriptomic datasets from many different angles to illustrate the roles of innate immune genes and nuclear stresses in promoting VSMC inflammation and the development of AAA and atherosclerosis. We made the following significant findings: *1)* innate immune genes from a new comprehensive list of innate immune regulators are significantly upregulated in the time courses of aortic diseases including atherosclerosis and aortic aneurysms; *2)* VSMCs are novel innate immune cells in response to DAMP stimulations by upregulating innate immune genes, cytokine and chemokine genes, plasma membrane protein genes and clusters of differentiation (CDs) markers (for adhesion and intercellular communications); *3)* genes encoding for proteins localized in nucleoli and membraneless stress organelles are upregulated in response to DAMPs as a novel nuclear stress response in addition to post-translational responses to nuclear stress (54); *4)* differential innate immune responses and nuclear stresses have been identified as novel atherosclerosis and aneurysm stage-specific and AAA model-specific mechanisms. Taken together, our study provided a novel insight for understanding the roles of innate immune responses and nuclear stresses in the development of AAA, atherosclerosis, and VSMC immunology and provided novel therapeutic targets for treating those significant cardiovascular and cerebrovascular diseases.

## Materials and methods

### Transcriptomic data and database content of human and mouse aortic disease and vascular smooth muscle cells

Transcriptomic datasets of human and mouse aortic diseases and VSMCs available in the public accessible NIH-NCBI Geodatasets database (https://www.ncbi.nlm.nih.gov/gds) were collected and organized. The transcription data analysis tool GEO2R in the NCBI-Geodatasets database was used for analysis. The datasets used for this study were the following: GSE10000 dataset: microarrays analysis of mouse aortic atherosclerotic lesions and adventitia in WT and apoE^–/–^ mice; GSE83500 dataset: microarray analysis of VSMCs isolated from human aortic wall atherosclerotic lesions in patients with myocardial infarction (MI); GSE57691 dataset: microarray analysis of aortic specimens obtained from aortic occlusive disease (AOD) patients and AAA patients; GSE17901 dataset: microarray analysis of suprarenal aorta of ApoE^–/–^ mice after ang II infusion for 7 days, 14 days, and 28 days. GSE147078 dataset: microarray analysis of mouse aorta after β-aminopropionitrile (BAPN) and Ang II infusion for 3 days; GSE51227 dataset: microarray analysis of infrarenal aortic tissue of a porcine pancreatic elastase (PPE)-induced AAA mouse model; GSE66280 dataset: microarray analysis of mouse aortic smooth muscle cells cultured with 4.5 g/l glucose; GSE47744 dataset: microarray analysis of mouse aortic VSMCs treated with 10 μg/mL cholesterol; GSE49519 dataset: microarray analysis of mouse aortic VSMCs from C57BL/6 and Signal Transducer and Activator of Transcription (STAT) 1 knockout mice with or without LPS stimulation; GSE9490 dataset: microarray analysis of human aortic VSMC treated with 0, 10 and 100 μmol/L DL-homocysteine; GSE11367 dataset: microarray analysis of human aortic VSMCs treated with 100 ng/ml IL-17 for 6 hours; GSE142417 dataset: microarray analysis of primary human aortic VSMCs stimulated with 5 ng/ml recombinant human transforming growth factor beta-1 (TGFβ1) for 24 hour; GSE30004 dataset: microarray analysis of human VSMCs treated with 1 ng/ml TGF-β; GSE180761 dataset: RNA-sequencing of human peripheral blood mononuclear cells (PBMCs) differentiated dendritic cells (DC) treated with Aryl hydrocarbon receptor (AHR) antagonist CH-223191-20 μM; GSE162015 dataset: RNA-sequencing of WT and NFKB1^–/–^ human macrophage induced THP-1 cell line either unstimulated or LPS-stimulated; GSE100671 dataset: microarray analysis of WT and NAPDH oxidase-2 deficient (NOX2)^–/–^ human myelomonoblastic cell line PLB-985; GSE29929 dataset: microarray analysis of liver tissue of WT and liver specific PERK knock-out (lsPERK^–/–^) mice with and without tunicamycin injection for 6 hours; GSE53038 dataset: microarray analysis of human embryonic stem cells (hESCs) treated with SET7 siRNA; GSE7810 dataset: microarray analysis of lungs type II cells from both NRF2^+/+^ and NRF2^–/–^ mice.

### Ingenuity pathway analysis and metascape analysis

Ingenuity pathway analysis (IPA, Qiagen, Redwood City, CA) was used to characterize the clinical relevance and molecular and cellular functions related to the genes in our RNA-seq data. Differentially expressed genes were collected and uploaded to IPA for further analysis. For the short upregulated gene lists that cannot be analyzed with IPA, the gene lists were analyzed with the Metascape website (https://metascape.org/gp/index.html#/main/step1) (**Figure 1A**).

### Python coding for gene filtering and processing

The data processing involves using the Pandas library in Python to convert the data into DataFrame format, filter genes based on specified conditions (e.g., p-value < 0.05, log2FC ≥ 1), and subsequently organize the resulting screened data into separate Excel files for subsequent statistical analysis (**Figure 1B**). Using Python to process data improves work efficiency and prevents potential errors in manual processing. Of note, it is essential to consider potential duplicate genes in certain datasets, where only data from the first occurrence of duplicates is considered valid.

## Results

### Innate immune pathways were upregulated in ApoE^–/–^ atherosclerotic aortas and ApoE^–/–^ Ang II-induced AAA

**Figure 2:**
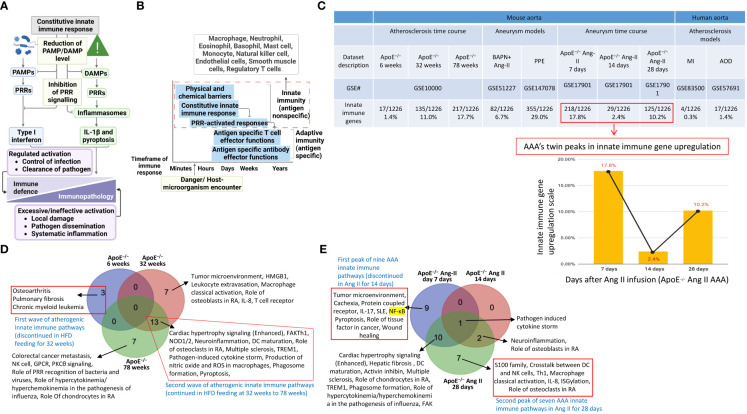
Innate immune genes upregulation in the aorta of atherogenic ApoE^–/–^ mice in high fat diet (HFD) feeding for 6 weeks, 32 weeks, and 78 weeks with two different innate immune waves and a twin-peak manner with the first peak at 7 days and the second peak at 28 days after angiotensin-II (Ang-II) infusion. **(A)** A schematic diagram revealed the constitutive and induced innate immune responses. **(B)** A schematic diagram illustrates the current understanding of innate immunity. **(C)** Innate immune genes were upregulated in 3 time-courses in the aorta of atherogenic apoE^–/–^ mice, 3 time-course in PAPN Ang-II and PPE-induced abdominal aortic aneurysm (AAA) mice, and 3 human atherosclerotic and AAA aortas. **(D)** Two different innate immune waves were identified in early (HFD for 6 weeks) with 3 pathways and advanced atherosclerosis (HFD for 32-78 weeks) with 13 pathways. **(E)** Two different innate immune peaks were also identified in early AAA (Ang-II infusion for 7 days) with nine innate immune pathways and late AAA (Ang-II infusion for 28 days) with seven innate immune pathways. PAMPs, pathogen-associated molecular pattern molecules; DAMPs, damage-associated molecular pattern molecules; PRRs, pattern recognition receptors; IL-1β, interleukin 1 beta; ApoE–/–, apolipoprotein E–deficient mouse; BAPN, beta-aminopropionitrile; Ang-II, angiotensin-II; PPE, porcine pancreatic elastase; MI, myocardial infarction; AOD, aortic occlusive disease; AAA, abdominal aortic aneurysm; MERS-CoV, Middle East respiratory syndrome coronavirus; HFD, high fat diet.

Our previous papers reported that the upregulated innate immune response genes play significant roles in endothelial cell activation and inflammation in response to viral infections such as COVID-19 homologous MERS-CoV, influenza virus, and sterile endogenous DAMPs such as oxidized low-density lipoprotein (oxLDL) (11) (**Figures 2A, B**), which verified our new working model that endothelial cells are innate immune cells (2, 3, 49). We also reported that the aorta in pathologies functions as an immune organ by upregulating a large number of secretomic genes (22). A complete list of innate immune genes (innatome) (11, 70) has never been examined to determine whether they are significantly upregulated in aortic diseases such as atherosclerosis and aortic aneurysms, with the exception of a short list of well-characterized cytokines and chemokines in aortic diseases (71, 72). We hypothesized that innate immune genes are significantly upregulated in aortic diseases. To examine this hypothesis, we collected 1226 genes out of 2308 innate immune genes after removing duplicated genes from the comprehensive innate immune database (*InnateDB*, https://www.innatedb.com/annotatedGenes.do?type=innatedb). The results in **Figure 2C** showed that: *a)* 1.4%, 11.0%, and 17.7% of innate immune genes were significantly upregulated in atherogenic ApoE^–/–^ aorta with HFD for 6 weeks, 32 weeks, 78 weeks, respectively; *b)* 6.7% and 29.0% of innate immune genes were upregulated in two aneurysm mouse models, including β-aminopropionitrile monofumarate (BAPN, an irreversible inhibitor of lysyl oxidase) (73)-Ang II-induced AAA and porcine pancreatic elastase (PPE)-induced AAA (74), respectively, suggesting that PPE-induced AAA induced the highest innate immune responses; *c)* upregulations of innate immune genes in ApoE^–/–^ Ang II-induced AAA model in three time points, including 7 days (17.8%), 14 days (2.4%), and 28 days (10.2%), which were in a twin-peak pattern; and *d)* By comparison, 0.3% to 1.4% of innate immune genes were upregulated in human atherosclerotic aorta and human aneurysm aorta, which were much lower than that in mouse models of aortic diseases;, suggesting that chronic vascular inflammation, such as atherosclerosis, displayed a distinct pattern of chronic innate immune responses with a continuous increase throughout 78 weeks, similar to what we reported in patients with CKD and end-stage renal disease (23), demonstrating that newly defined chronic innate immune responses may not be in remission as prototypic acute innate immune responses (**Figure 2B**).

Of note, since there were a lot of data throughout the analyses that cannot be included in a limited space in the manuscript, our attention was focused on identifying the differences between: *i)* the time course of atherosclerosis; *ii)* the time course of AAA; *iii*) different models of AAA; and i*v)* different stimuli-induced expression changes of VSMCs. The ingenuity pathway analysis (IPA) results showed that *1)* three upregulated pathways, including osteoarthritis, pulmonary fibrosis idiopathic, and chronic myeloid leukemia, in ApoE^–/–^ at 6 weeks were discontinued; instead, 13 upregulated pathways, including cardiac hypertrophy, NOD1/2 neuroinflammation, Th1-dendritic cell maturation, osteoclasts in rheumatoid arthritis, multiple sclerosis, pathogen-induced cytokine storm, TEM1, nitric oxide and ROS in macrophages, phagosome formation, pyroptosis, and FAK, were continued through ApoE^–/–^ at 32 weeks to 78 weeks (**Figure 2D**), suggesting that atherosclerosis has two-wave inflammation with the first wave at 6 weeks of HFD and the second wave from 32 weeks continuing to 78 weeks of HFD; and the 13 pathways were conserved in advancing atherosclerotic inflammation; *2)* 9 upregulated pathways were found specific in ApoE^–/–^ Ang II at 7 days, including tumor microenvironment, cachexia, protein coupled receptor, IL-17, SLE, NF-KB, pyroptosis, role of tissue factor in cancer, and wound healing; 7 upregulated pathways were found specific in ApoE^–/–^ Ang II at 28 days, including S100 family, crosstalk between DC and NK Cells, Th1, macrophage classical activation, IL-8, ISGylation, and role of osteoclasts in rheumatoid arthritis; but no upregulated pathway was found specific in ApoE^–/–^ Ang II at 14 days (**Figure 2D**); and *3)* Several pathways were upregulated in human myocardial infarction, AAA, and aortic occlusive diseases (**Supplementary Figures S1A–C**).

**Figure 3:**
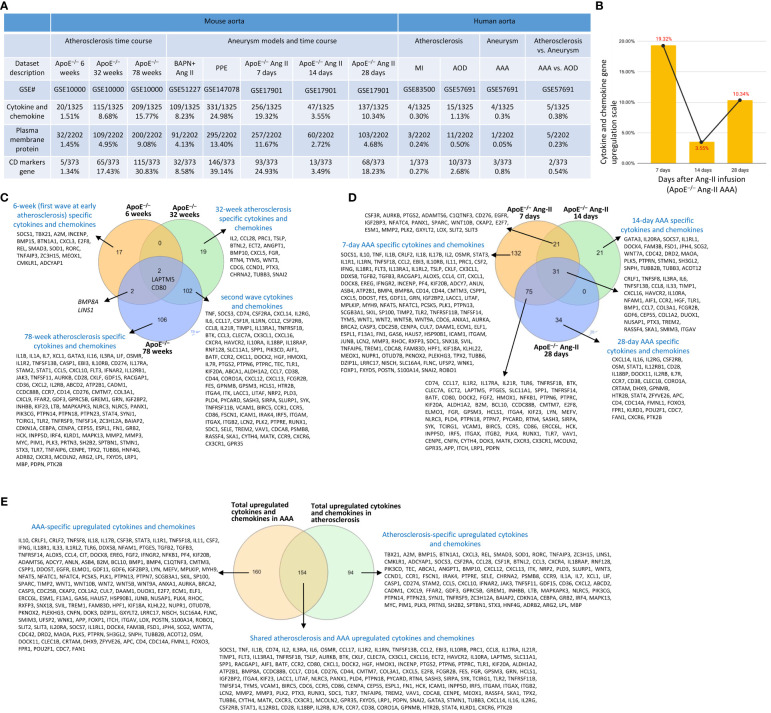
Atherosclerosis and abdominal aortic aneurysm (AAA) stage-specific cytokines and chemokines, plasma membrane proteins, and CD markers identified in ApoE^–/–^ high fat diet (HFD) for 6, 32 and 78 weeks and ApoE^–/–^ angiotensin II (Ang-II) infusion for 7, 14 and 28 days, respectively. **(A)** Cytokines and chemokines, plasma membrane proteins, and CD marker genes were upregulated in the time course of atherosclerosis in ApoE^–/–^ aorta and aortas of three mouse models of AAA, including BAPN+Ang II-induced AAA, PPE-induced AAA and 3 time course of ApoE^–/–^ Ang II-induced AAA and human samples of aortic diseases. **(B)** The percentage of upregulated cytokine and chemokine genes confirmed twin-peak inflammation pattern in the time course of AAA. **(C)** Atherosclerosis stage-specific cytokines and chemokines identified at HFD for 6, 32 and 78 weeks. **(D)** AAA stage-specific cytokines and chemokines identified in Ang-II infusion for 7, 14 and 28 days. **(E)** 154 AAA-specific upregulated cytokines and chemokines, 94 atherosclerosis-specific upregulated cytokines and chemokines, and 160 AAA and atherosclerosis shared cytokines and chemokines. Cytokines and chemokines, plasma membrane protein, CD markers gene lists were collected from the Human Protein Atlas (HPA) database (https://www.proteinatlas.org). ApoE^–/–^, apolipoprotein E–deficient mouse; BAPN, beta-aminopropionitrile; Ang II, angiotensin-II; PPE, porcine pancreatic elastase; MI, myocardial infarction; AOD, aortic occlusive disease; AAA, abdominal aortic aneurysm.

To further examine secreted/soluble molecules of innate immune responses, we collected a comprehensive list of 1325 cytokines and chemokines from the Human Protein Atlas (HPA) database (https://www.proteinatlas.org/search/cytokine; https://www.proteinatlas.org/search/chemokine). As shown in **Figure 3A**, *a)* 1.51%, 8.68%, and 15.77% of cytokine and chemokine genes were significantly upregulated in ApoE^–/–^ aorta with HFD at 6 weeks, 32 weeks, and 78 weeks, respectively; *b)* 8.23% and 24.98% of cytokine and chemokine genes were upregulated in two aneurysm mouse models, including BAPN-Ang II-induced AAA and PPE-induced AAA, respectively, suggesting that PPE-induced AAA induced the highest cytokine and chemokine responses; *c)* upregulations of cytokine and chemokine genes in ApoE^–/–^ Ang II-induced AAA in three time points, including 7 days (19.32%), 14 days (3.55%), and 28 days (10.34%), which were in a twin-peak pattern (**Figure 3B**); *d)* By comparison, 0.3% to 1.3% of cytokine and chemokine genes were upregulated in human atherosclerotic aorta and human aneurysm aorta, respectively, which were much lower than that in mouse models of aortic diseases.

To further examine intercellular communication and intercellular interaction mediators of innate immune responses as we reported (75, 76), we collected a comprehensive list of 2202 plasma membrane protein genes from the HPA database (https://www.proteinatlas.org/search/subcell_location%3APlasma+membrane%2CCell+Junctions) (77) and a list of 373 clusters of differentiation (CDs) (75). Surprisingly, 124 (33%) out of 373 CD markers overlapped with those of plasma membrane protein genes, suggesting that 67% of CDs may have multiple subcellular membrane locations, as we reported (58). As shown in **Figure 3A**, *a)* 1.45%, 4.95%, and 9.08% of plasma membrane protein genes, 1.34%, 17.43%, and 30.83% of CD marker genes were significantly upregulated in ApoE^–/–^ aorta with HFD at 6 weeks, 32 weeks, and 78 weeks, respectively; *b)* 4.13% and 13.4% of plasma membrane protein genes, 8.58% and 39.14% of CD marker genes were upregulated in two aneurysm mouse models, including BAPN-Ang II-induced AAA and PPE-induced AAA, respectively, suggesting that PPE-induced AAA model induced the highest plasma membrane protein genes and CD marker genes responses among AAA models; *c)* upregulations of plasma membrane protein and CD marker genes in ApoE^–/–^ Ang II-induced AAA in three time points, including 7 days (11.67% and 24.93%), 14 days (2.72% and 3.49%), and 28 days (4.68% and 18.23%), respectively, which were in a twin-peak pattern. Of note, the upregulation scales of cytokines (15.77%), plasma membrane protein genes (9.08%), and CD marker genes (30.83%) in ApoE^–/–^ HFD at 78 weeks were similar to those of cytokines (19.32%), plasma membrane protein genes (11.67%), and CD marker genes (24.93%) in ApoE^–/–^ Ang II at 7 days, suggesting that Ang II in ApoE^–/–^ HFD background significantly accelerates and shortens the inflammation upregulation process by 7 – 8 times. Venn diagram analysis of ApoE^–/–^HFD time course in **Figure 3C** showed upregulation of 17 cytokines and chemokines in ApoE^–/–^ at 6 weeks, representing the first wave cytokines and chemokines, 19 atherosclerosis-specific cytokines and chemokines at 32 weeks, 102 second wave cytokines and chemokines were shared between 32 and 78 weeks; however, 78 weeks showed 106 atherosclerosis-specific cytokines and chemokines. Of note, atherosclerosis-stage-specific cytokines and chemokines were not reported in the original report (78). Venn diagram analysis of ApoE^–/–^ Ang II-induced AAA time course showed 132 at 7 days AAA-specific cytokines and chemokines (AAA stage-specific cytokines and chemokines were not reported in the original paper (79), 21 at 14 days AAA-specific cytokines and chemokines, and 34 at 28 days AAA-specific cytokines and chemokines (**Figure 3D**). Furthermore, 160 ApoE^–/–^ Ang II AAA-stage-specific cytokines and chemokines have also been identified at Ang II infusion for 7 days, 14 days, and 28 days, and 94 atherosclerosis stage-specific cytokines and chemokines have been identified at HFD feeding for 6 weeks, 32 weeks, and 78 weeks (**Figure 3E**).

### Nuclear membrane genes, nucleoli genes, and nucleoplasm genes were upregulated in ApoE^–/–^ atherosclerotic aortas and ApoE^–/–^ Ang II-induced AAA

**Figure 4:**
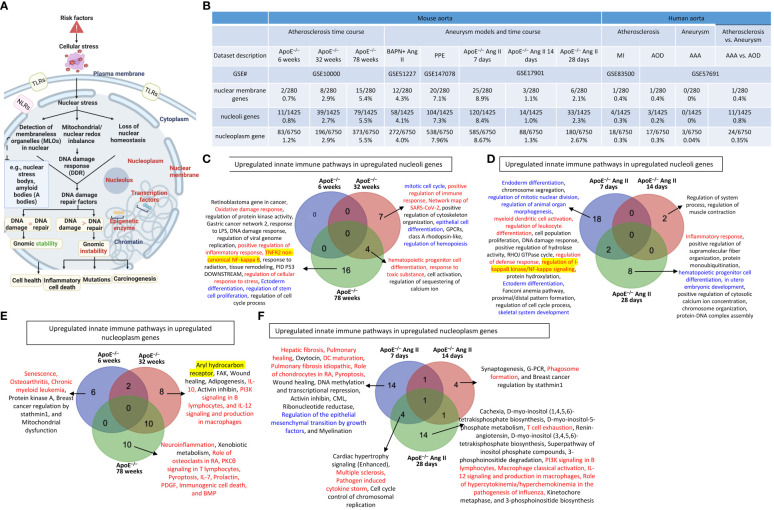
Nuclear membrane protein genes, nucleoli protein genes, and nucleoplasm proteins genes upregulation in the time course of atherosclerosis in ApoE^–/–^ aorta and aortas of three mouse models of AAA including, BAPN + Ang-II, PPE and 3 time course of ApoE^–/–^ Ang-II AAA and human samples of aortic diseases. **(A)** Nuclear stress sensing acts as an integrative part of the cellular organelle sensing system and plays an important role in bridging disease risk factors and the innate immune responses. The expression changes of three nucleus localized protein lists were used as readouts for nuclear stresses in aortic pathologies. **(B)** Nuclear membrane genes, nucleoli genes and nucleoplasm genes were upregulated in the time course of atherosclerosis in ApoE^–/–^ aorta and aortas of three mouse models of AAA and 3 time course of ApoE^–/–^ Ang-II AAA and human samples of aortic diseases. **(C)** Several innate immune pathways (in red) and differentiation pathways (in blue) were upregulated in nucleoli genes in ApoE^–/–^ atherosclerosis 32 weeks and 78 weeks. **(D)** Several innate immune pathways (in red), differentiation pathways (in blue), and master gene pathways (highlighted in yellow) were upregulated in nucleoli genes in ApoE^–/–^ Ang-II 7 days and 28 days. **(E)** Several innate immune pathways (in red) and master gene pathway (highlighted in yellow) were upregulated in nucleoplasm genes in ApoE^–/–^ atherosclerosis 6 weeks, 32 weeks and 78 weeks. **(F)** Upregulated innate immune pathways in upregulated nucleoplasm genes in ApoE^–/–^ Ang-II AAA 7 days were significantly different from that of ApoE^–/–^ Ang-II AAA 14 days and that of 28 days. 280 nuclear membrane genes were collected from the nuclear membrane gene database in the HPA database (https://www.proteinatlas.org/humanproteome/subcellular/nuclear+membrane), 1425 nucleoplasm genes were collected from the nucleoli gene database in the HPA database (https://www.proteinatlas.org/humanproteome/subcellular/nucleoli), 6753 nucleoplasm genes were collected from the nucleoplasm gene database in the HPA database (https://www.proteinatlas.org/humanproteome/subcellular/nucleoplasm). TLRs, Toll-like receptors; NLRs, nucleotide-binding oligomerization domain-like receptors; ApoE^–/–^, apolipoprotein E–deficient mouse; BAPN, beta-aminopropionitrile; Ang II, angiotensin II; PPE, porcine pancreatic elastase; MI, myocardial infarction; AOD, aortic occlusive disease; AAA, abdominal aortic aneurysm.

It has been reported that nucleoli are a nuclear stress response membraneless organelle (63–65). Single-cell measurement of nuclear proteins and RNAs *in vivo* was recently developed (80). However, global nuclear stresses in CVDs remain poorly characterized. We hypothesized that transcriptomic reprogramming of nuclear membrane proteins, nucleoli proteins, and nucleoplasm proteins is modulated as a novel read-out for nuclear stresses (**Figure 4A**). To examine this hypothesis, we collected three complete lists of 280 nuclear membrane proteins (NMPs), 1425 nucleoli proteins (NPs), and 6750 nucleoplasm proteins (NPPs) from the comprehensive HPA database (https://www.proteinatlas.org/). The results showed that: *a)* 0.7% of NMPs, 0.8% of NPs, 1.2% of NPPs in ApoE^–/–^ HFD at 6 weeks, 2.9% of NMPs, 2.7% of NPs, 2.9% of NPPs in ApoE^–/–^ HFD at 32 weeks, 5.4% of NMPs, 5.5% of NPs, 5.5% of NPPs in ApoE^–/–^ HFD at 78 weeks were significantly upregulated, respectively; *b)* 4.3% of NMPs, 4.1% of NPs, 4.0% of NPPs in BAPN Ang II-induced AAA, 7.1% of NMPs, 7.3% of NPs, 7.96% of NPPs in PPE-induced AAA were significantly upregulated, respectively, suggesting that nuclear stresses induced in BAPN Ang II-induced AAA and PPE-induced AAA were higher than that of ApoE^–/–^ Ang II-induced AAA; *c)* upregulations of nuclear stress genes in ApoE^–/–^ Ang II-induced AAA model in three time points, including at 7 days (8.9% NMPs, 8.4% NPs, 8.67% NPPs), at 14 days (1.1% NMPs, 1.0% NPs, 1.3% NPPs) and at 28 days (2.1% NMPs, 2.3% NPs and 2.67% NPPs), which were in a twin-peak pattern; *and d)* By comparison, 0% to 0.8% of nuclear stress genes were upregulated in human atherosclerotic aorta and human aneurysm aorta, which were much lower than that in mouse models of aortic diseases (**Figure 4B**). The upregulated nuclear membrane protein genes in ApoE^–/–^ HFD at 6 weeks, 32 weeks, and 78 weeks, ApoE^–/–^ Ang II-AAA at 7days, 14 days, and 28 days, human MI, human AAA, and human AOD were shown in the **Supplementary Figures S2A–E**. The upregulated nucleoli genes in ApoE^–/–^ HFD at 6 weeks, 32 weeks, and 78 weeks, ApoE^–/–^ Ang II-AAA at 7days, 14 days, and 28 days, human MI, human AAA, and human AOD were shown in the **Supplementary Figures S2F–J**. However, the upregulated nucleoplasm genes in human MI, human AAA, and human AOD were shown in the **Supplementary Figures S2K–M**.

In analyzing upregulated nucleoli pathways by the Metascape we found that no specific pathways in ApoE^–/–^ at 6 weeks; 7 upregulated nucleoli pathways specific in ApoE^–/–^ at 32 weeks, including 2 innate immune pathways (highlighted in red) such as positive regulation of immune response and network map of SARS-CoV-2 and 3 differentiation pathways (highlighted in blue) such as mitotic cell cycle, epithelial cell differentiation, and regulation of hemopoiesis; 16 upregulated nucleoli pathways specific in ApoE^–/–^ at 78 weeks, including 4 innate immune pathways (highlighted in red) such as oxidative damage response, positive regulation of inflammatory response, TNFR2 non-canonical NF-kB, and regulation of cellular response to stress, and one master transcription factor (highlighted in yellow) such as TNFR2 non-canonical NF-kappa B; 4 upregulated nucleoli genes shared between ApoE^–/–^ at 32 and 78 weeks, including 2 innate immune pathways (highlighted in red) such as hematopoietic progenitor cell differentiation and response to toxic substance (**Figure 4C**); 18 pathways were specific in ApoE^–/–^ Ang II at 7 days, including 4 innate immune pathways (highlighted in red) such as myeloid dendritic cell activation, regulation of leukocyte differentiation, regulation of defense response, and regulation of I-kappaB kinase/NF-kappaB (NF-kB) signaling, five differentiation pathways (highlighted in blue) such as endoderm differentiation, regulation of mitotic nuclear division, regulation of animal organ morphogenesis, ectoderm differentiation, and skeletal system development, and one master transcription factor (highlighted in yellow) such as regulation of I-kappaB kinase/NF-kappaB (NF-kB) signaling; 2 pathways were found specific in ApoE^–/–^ Ang II at 14 days; 8 upregulated pathways were found specific in ApoE^–/–^ Ang II at 28 days, including 1 innate immune pathway (highlighted in red) such as inflammatory response, and differentiation pathways (highlighted in blue) such as hematopoietic progenitor cell differentiation and *in utero* embryonic development (**Figure 4D**).

In analyzing upregulated nucleoplasm pathways by IPA, we found that: *1)* 6 upregulated pathways were found specific in ApoE^–/–^ at 6 weeks including 3 innate immune pathways (highlighted in red) such as senescence, osteoarthritis, and chronic myeloid leukemia; 8 upregulated pathways were found specific in ApoE^–/–^ at 32 weeks including 3 innate immune pathways (highlighted in red) such as IL-10, PI3K signaling in B lymphocytes, and IL-12 signaling and production in macrophages, and one master gene (highlighted in yellow) such as aryl hydrocarbon receptor; and 10 upregulated pathways were found specific in ApoE^–/–^ at 78 weeks including 8 innate immune pathways (highlighted in red) such as neuroinflammation, role of osteoclasts in rheumatoid arthritis, PKCθ signaling in T lymphocytes, pyroptosis, IL-7, prolactin, PDGF, immunogenic cell death, and BMP (**Figure 4E**); 14 upregulated pathways were found specific in ApoE^–/–^ Ang II at 7 days including 6 innate immune pathways (highlighted in red) such as hepatic fibrosis, pulmonary healing, dendritic cell maturation, pulmonary fibrosis idiopathic, role of chondrocytes in rheumatoid arthritis, and pyroptosis, and one differentiation pathway (highlighted in blue) such as regulation of the epithelial mesenchymal transition by growth factors; 4 upregulated pathways were found specific in ApoE^–/–^ Ang II at 14 days including one innate immune pathway (highlighted in red) such as phagosome formation; and 14 upregulated pathways were found specific in ApoE^–/–^ Ang II at 28 days including 5 innate immune pathways (highlighted in red) such as T cell exhaustion, PI3K signaling in B lymphocytes, macrophage classical activation, IL-12 signaling and production in macrophages, and role of hypercytokinemia/hyperchemokinemia in the pathogenesis of influenza (**Figure 4F**).

### Transcription factor pathways were upregulated in ApoE^–/–^ atherosclerotic aortas and ApoE^–/–^ Ang II-induced AAA as well as in PPE-AAA and BAPN Ang II- AAA

**Figure 5:**
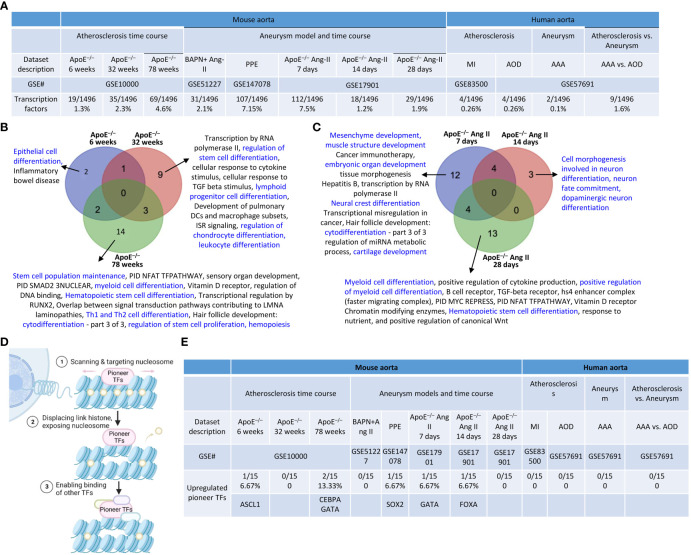
Transcription factor (TFs) genes upregulation in the time course of atherosclerosis in ApoE^–/–^ aorta and aortas of three mouse models of AAA and 3 time course of ApoE^–/–^ Ang-II AAA and human samples of aortic diseases. **(A)** The time course changes of the upregulated TFs in ApoE^–/–^ Ang-II exhibited a twin peak pattern of transcription reprogramming, characterized by a prominent peak at 7 days followed by a smaller peak at 28 days. **(B)** Several differentiation pathways (in blue) in upregulated TFs were upregulated in ApoE^–/–^ atherosclerosis time course. **(C)** Several differentiation pathways (in blue) in upregulated TFs were upregulated in ApoE ^–/–^ Ang II AAA time course. **(D)** Pioneer TFs are a special group of TFs that their binding to regulatory regions is the first event in gene transcription and can occur in silent or heterochromatin regions. **(E)** Five pioneer TFs, including ASCL1, CEBPA, GATA, SOX2, and FOXA, play significant role in aortic diseases. 1496 TFs were collected from the HPA database (https://www.proteinatlas.org/search/protein_class:Transcription+factors). 15 pioneer TFs were collected PMID: 29507097. ApoE^–/–^, apolipoprotein E–deficient mouse; BAPN, beta-aminopropionitrile; Ang-II, angiotensin-II; PPE, porcine pancreatic elastase; MI, myocardial infarction; AOD, aortic occlusive disease; AAA, abdominal aortic aneurysm.

We previously reported that transcription factors (TFs) GATA3, HDAC6, and Bcl-6 regulate Treg plasticity and determine Treg conversion into either novel antigen-presenting cell-like Treg or type 1 T helper cell (Th1)-Treg (21), suggesting that non-Treg T helper cell subsets such as Th2 TF GATA3, follicular T helper cell (Tfh) TF Bcl-6 and HDAC6 cooperate with Treg-specific TF Foxp3 to determine Treg transcriptomes and functions. We hypothesized that aortic diseases upregulate specific TFs. To test this hypothesis, we collected 1496 TFs from a comprehensive HPA database (https://www.proteinatlas.org/search/protein_class:Transcription+factors) as we reported recently (11, 23–25, 75, 77, 81). As shown in **Figure 5A**, we found that: 1) 19, 35, and 69 TFs were upregulated in ApoE^–/–^ at 6 weeks, 32 weeks, and 78 weeks, respectively; 2) 31 and 107 TFs were upregulated in BAPN Ang II-induced AAA and PPE-induced AAA, respectively; 3) 112, 18, and 29 TFs were upregulated in ApoE^–/–^ Ang II-induced AAA at 7 days, 14 days, and 28 days, respectively, also indicating the twin-peak pattern as found in innate immune genes, nuclear membrane genes, nucleoli genes, and nucleoplasm genes; and 4) TF expressions were also upregulated in 0.1% to 1.6% in four human aorta samples but on much smaller scales than that in mouse aortas. The upregulated TFs in human MI, AAA, and AOD were shown in the **Supplementary Figures S3A–C**.

The Metascape pathway analysis of upregulated TFs showed that: *1)* 2 upregulated pathways were found specific in ApoE^–/–^ at 6 weeks including a differentiation pathway (highlighted in blue) such as epithelial cell differentiation; 9 upregulated pathways were found specific in ApoE^–/–^ 32 weeks including 4 differentiation pathways (highlighted in blue) such as regulation of stem cell differentiation, lymphoid progenitor cell differentiation, regulation of chondrocyte differentiation, and leukocyte differentiation; 14 upregulated pathways were found specific in ApoE^–/–^ 78 weeks including 7 differentiation pathways (highlighted in blue) such as stem cell population maintenance, myeloid cell differentiation, hematopoietic stem cell differentiation, Th1 and Th2 cell differentiation, cytodifferentiation, regulation of stem cell proliferation, and hemopoiesis; suggesting that TF pathways in three different stages of atherosclerosis progression in ApoE^–/–^ HFD aortas are significantly different (**Figure 5B**). 12 TFs upregulated pathways were found specific in ApoE^–/–^ Ang II at 7 days including 6 differentiation pathways (highlighted in blue) such as mesenchyme development, muscle structure development, embryonic organ development, neural crest differentiation, cytodifferentiation, and cartilage development; 3 TFs upregulated pathways were found specific in ApoE^–/–^ Ang II at 14 days, including 3 differentiation pathways (highlighted in blue) such as cell morphogenesis involved in neuron differentiation, neuron fate commitment, dopaminergic neuron differentiation; 13 upregulated pathways were found specific in ApoE^–/–^ Ang II at 28 days including 3 differentiation pathways (highlighted in blue) such as myeloid cell differentiation, positive regulation of myeloid cell differentiation, and hematopoietic stem cell differentiation (**Figure 5C**), suggesting that TF pathways in three different stages of AAA progression in ApoE^–/–^ Ang II aorta model are significantly different.

Pioneer transcription factors are a special group of TFs whose their binding to regulatory regions is the first event in gene transcription and can occur in silent or heterochromatin regions (82) (**Figure 5D**). We hypothesized that pioneer TF upregulation is a feature of aortic disease progression. Therefore, we examined 15 pioneer TFs (83) and found that one (ASCL1) and two (CEBPA and GATA) pioneer TFs were upregulated in ApoE^–/–^ at 6 weeks and 78 weeks, respectively; one (SOX2) pioneer TFs was upregulated in PPE-AAA; and one (GATA) and one (FOXA) pioneer TFs were upregulated in ApoE^–/–^ Ang II-AAA at 7 days and 14 days, indicating that five pioneer TFs, including ASCL1, CEBPA, GATA, SOX2, and FOXA, play significant role in aortic diseases (**Figure 5E**). These results demonstrated that upregulation of pioneer TFs in atherosclerosis and aortic aneurysms are disease-stage-specific events.

### VSMCs are innate immune cells as judged by the upregulation of innate immune genes, cytokine and chemokine genes, and plasma membrane proteins and CD marker genes

**Figure 6:**
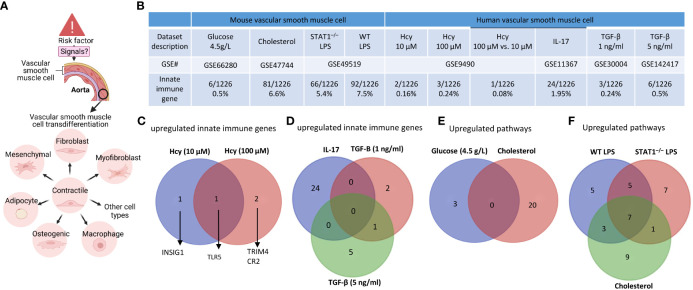
Innate immune gene upregulation in mouse and human vascular smooth muscle cells (VSMCs) stimulated with high glucose, cholesterol, lipopolysaccharides (LPS), homocysteine (Hcy), interleukin-17 (IL-17), and transforming growth factor-B (TGF-B). **(A)** VSMCs phenotype switching (trans-differentiation) under disease conditions into mesenchymal-like, fibroblast-like, myofibroblast-like, macrophage-like, osteogenic-like, and adipocyte-like VSMCs. **(B)** Innate immune genes were significantly upregulated in mouse VSMCs treated by cholesterol and LPS, to a greater extent than that in human VSMCs under atherogenic conditions. **(C)** One upregulated innate immune gene was found specific in Hcy (10 µM) and 2 upregulated genes were found specific in Hcy (100 µM) treated VSMCs. **(D)** 24, 2, and 5 upregulated innate immune genes were found specific in IL-17, TGF-B (1 ng/ml), and TGF-B (5 ng/ml) treated VSMCs, respectively; and 1 upregulated gene was found overlapped between IL-17 and TGF-B (1 ng/ml) treated VSMCs. **(E)** 3 upregulated pathways were found specific in glucose (4.5 g/L) cultured VSMCs, and 20 upregulated pathways were found specific in cholesterol loaded VSMCs. **(F)** 5, 7, and 9 upregulated pathways were found specific in WT LPS treated, STAT^–/–^ LPS treated, and cholesterol loaded VSMCs, respectively, 5 upregulated pathways were found overlapped between WT LPS treated and STAT^–/–^ LPS treated VSMCs, 1 upregulated pathway was found overlapped between STAT^–/–^ LPS treated and cholesterol loaded VSMCs, 3 upregulated pathways were found overlapped between cholesterol loaded and WT LPS treated VSMCs, 7 upregulated pathways were found overlapped between WT LPS treated, STAT^–/–^ LPS treated and cholesterol loaded VSMCs. 1226 out of 2308 Innate Immunity genes were collected from the innate immunity genes database in InnateDB after removing duplicate genes (https://www.innatedb.com/annotatedGenes.do?type=innatedb).

Based on those findings outlined in the introduction, we hypothesized that innate immune responses play significant roles in contributing to VSMC trans-differentiation and phenotypic switching in aortic diseases and VSMCs in pathologies are innate immune cells and contribute to aortic diseases, as cellular mechanisms (**Figure 6A**). As shown in **Figure 6B**, innate immune genes were upregulated in VSMCs stimulated by proinflammatory DAMPs (2, 33) such as high glucose (84), cholesterol, and proinflammatory pathogen-associated molecular pattern (PAMP) lipopolysaccharide (LPS, an endotoxin from gram-negative bacteria) stimulated signal transducer and activator of transcription 1 (STAT1)^–/–^ VSMCs and LPS stimulated wild-type (WT) VSMCs (85), respectively. In addition, innate immune genes were upregulated in human VSMCs stimulated by metabolic DAMP (86, 87) homocysteine (Hcy) (10 μM), Hcy (100 μM), Hcy (100 μM) versus Hcy (10 μM) (87–89), proinflammatory cytokine IL-17 (49, 50), anti-inflammatory (90, 91), Treg-inducing (21), aneurysm-suppressing (92) and VSMC senescence/Marfan syndrome-inducing (93) cytokine TGF-β (1 ng/ml), and TGF-β (5 ng/ml), respectively. The upregulated innate immune genes in VSMCs stimulate with Hcy, IL-17, TGF-β, cholesterol, and glucose were shown in the **Supplementary Figures S4A–F**.

The IPA analysis and gene analysis showed that: one upregulated innate immune gene was found specific in Hcy (10 µM)-treated VSMCs including lipid metabolism regulator insulin induced gene 1 (INSIG1) (94); 2 upregulated genes were found specific in Hcy (100 µM)-treated VSMCs, including mitochondrial interacting RING E3 ligase TRIM4 (95) and CR2; one upregulated gene was found to overlap between Hcy (10 µM) and Hcy (100 µM)-treated VSMCs including inflammation mediator Toll-like receptor 5 (TLR5) (96) (**Figure 6C**); 24 upregulated innate genes in IL-17-treated VSMCs were different from 8 innate immune genes in TGF-β-treated VSMCs (**Figure 6D**); 3 pathways were upregulated in VSMCs stimulated by high glucose such as positive regulation of catabolic process, response to hormone, and negative regulation of cell differentiation; 20 pathways were upregulated in cholesterol-loaded VSMCs, including TLR, regulation of inflammatory response, immune effector process, Legionellosis, cytokine signaling, response to type II interferon, lung fibrosis, regulation of DNA-binding transcription factor activity, positive regulation of cytokine production, leukocyte activation, response to virus, innate immune response, TNF, IL-18, positive regulation of response to external stimulus, defense response to bacterium, regulation of leukocyte activation, TLR cascades, response to bacterium, and regulation of response to cytokine stimulus (**Figure 6E**); 5 pathways were specifically upregulated in LPS-stimulated VSMCs, including regulation of defense response, interferon, non-genomic actions of 1,25 dihydroxyvitamin D3, regulation of type I interferon production, SARS-CoV-2 innate immunity evasion, and cell-specific immune response; 7 pathways were specifically upregulated in LPS-stimulated STAT1^–/–^ VSMCs, including NOD-like receptor, lipid and atherosclerosis, cellular response to virus, Kaposi sarcoma-associated herpesvirus infection, TLR4 cascade, cellular response to cytokine stimulus, and apoptosis; and 9 pathways were specifically upregulated in cholesterol-loaded VSMCs, including regulation of inflammatory response, immune effector process, leukocyte activation, IL-18, positive regulation of response to external stimulus, Legionellosis, lung fibrosis, TLR cascades, and response to bacterium (**Figure 6F**).

**Figure 7:**
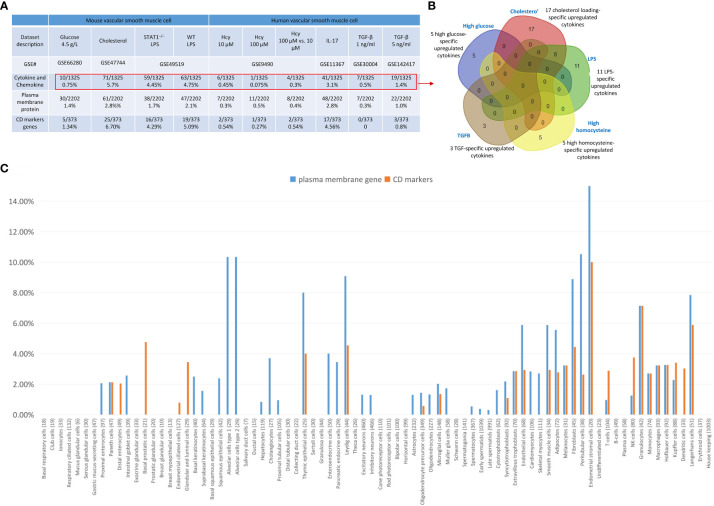
Upregulated cytokine and chemokine, plasma membrane protein genes and CD marker genes in VSMCs stimulated by aortic disease conditions indicated the trans-differentiation potentials of VSMCs into 30 new cell types and 23 new cell types, respectively. **(A)** Cytokine and chemokine genes, plasma membrane protein genes, and CD marker genes were significantly upregulated in mouse vascular smooth muscle cells (VSMCs) and human VSMCs under atherogenic conditions, such as high glucose, cholesterol, lipopolysaccharides (LPS), homocysteine (Hcy), interleukin-17 (IL-17), and transforming growth factor-B (TGF-B). **(B)** Stimulation-specific upregulated cytokines identified among total upregulated cytokines and chemokines in VSMCs. **(C)** 8065 markers of human 79 cell types were collected from the HPA database (https://www.proteinatlas.org/humanproteome/tissue+cell+type). The percentage is obtained by dividing the number of upregulated plasma membrane protein or CD marker genes by the total number of markers for the specific cell type. Cytokines and chemokines, plasma membrane, and CD markers were collected from the HPA database (https://www.proteinatlas.org).

Furthermore, we examined cytokines and chemokines (**Figure 7A**) and found that 0.75%, 5.7%, 4.45%, and 4.75% of cytokine and chemokine genes were upregulated in VSMCs stimulated by high glucose, cholesterol, STAT1^–/–^ LPS-stimulated VSMCs, and WT LPS-stimulated VSMCs, respectively. In addition, 0.45%, 0.075%, 0.3%, 3.1%, 0.5%, and 1.4% of cytokine and chemokine genes were upregulated in human VSMCs stimulated by Hcy (10 μM), Hcy (100 μM), Hcy (100 μM) versus (10 μM), IL-17, TGF-β (1 ng/ml), and TGF-β (5 ng/ml), respectively. Venn diagram analysis in **Figure 7B** showed *i)* 5 high glucose-specific upregulated cytokines, including *ITGA5*, *ENPP1*, *MCAM*, *CD248*, and *ITGA1*; *ii)* 17 cholesterol loading-specific upregulated cytokines, including *CDH5*, *IL2RG*, *CD68*, *TEK*, *CD93*, *ACKR1*, *ICAM2*, *CD302*, *ENPP3*, *CD200*, *BST2*, *TNFRSF9*, *BST*, *CXCR4*, *S1PR1*, and *IL13RA2*; *iii)* 11 LPS-specific upregulated cytokines, including *CD74, CD44, ICAM1, TLR3, PVR, SELP, IL15RA, GYPC, ICAM4, CD47*, and *VCAM1*; *iv)* 5 high homocysteine-specific upregulated cytokines, including *PROM1, NCAM1, RHAG, CR2*, and *BMPR1b*; and *v)* 3 TGF-specific upregulated cytokines, including *CSF1R*, *SEMA7A*, and *SDC1*, suggesting that stimulation-specific upregulated cytokines were identified among total upregulated cytokines in VSMCs.

We also examined 2202 plasma membrane proteins from the HPA database (77) and a list of 373 CD markers (75). Surprisingly, 124 (33%) out of 373 CD markers overlapped with those of plasma membrane proteins, suggesting that 67% of CD markers may have multiple subcellular membrane locations. As shown in **Figure 7A**, we found that 1.4%, 2.8%, 1.7%, and 2.1% of plasma membrane protein genes were upregulated in VSMCs stimulated by high glucose, cholesterol, STAT1^–/–^ LPS-stimulated VSMCs and WT LPS-stimulated VSMCs, respectively. In addition, 1.34%, 6.7%, 4.29%, and 5.09% of CD marker genes were upregulated in VSMCs stimulated by high glucose, cholesterol, STAT1^–/–^ LPS-stimulated VSMCs and WT LPS-stimulated VSMCs, respectively. Moreover, 0.3%, 0.5%, 0.4%, 2.8%, 0.3%, and 1.0% of plasma membrane protein genes were upregulated in human VSMCs stimulated by Hcy (10 μM), Hcy (100 μM), Hcy (100 μM) versus Hcy (10 μM), IL-17, TGF-β (1 ng/ml), and TGF-β (5 ng/ml), respectively. Furthermore, 0.54%, 0.27%, 0.54%, 4.56%, 0%, and 0.8% of CD marker genes were upregulated in human VSMCs stimulated by Hcy (10 μM), Hcy (100 μM), Hcy (100 μM) versus Hcy (10 μM), IL-17, TGF-B (1 ng/ml), and TGF-B (5 ng/ml), respectively. These results demonstrated that in atherosclerotic and aneurysm conditions, CD marker genes are upregulated more than plasma membrane protein genes in VSMCs.

Next, we examined the upregulated CD marker genes and plasma membrane protein genes in VSMCs stimulated by aortic disease conditions in 8065 markers of human 79 cell types identified in the tissue cell type section in the HPA database (https://www.proteinatlas.org/humanproteome/tissue+cell+type) (**Figure 7C**) and found that 18 cell types exhibit a proportion of upregulated plasma membrane protein genes within the cell-type markers that ranges from 2% to less than 5%, including proximal enterocytes, paneth cells, intestinal goblet cells, basal keratinocytes, squamous epithelial cells, cholangiocytes, enteroendocrine cells, pancreatic endocrine cells, microglial cells, syncytiotrophoblasts, extravillous trophoblasts, cardiomyocytes, skeletal myocytes, melanocytes, monocytes, macrophages, Hofbauer cells, Kupffer cells; 12 cell types exhibit a proportion of upregulated plasma membrane protein genes within the cell-type markers that equal to or greater than 5%, including alveolar cells type 1, alveolar cells type 2, thymic epithelial cells, Leydig cells, endothelial cells, smooth muscle cells, adipocytes, fibroblasts, peritubular cells, endometrial stromal cells, granulocytes, Langerhans cells; 20 cell types exhibit a proportion of upregulated CD marker genes within the cell-type markers that ranges from 2% to less than 5%, including paneth cells, distal enterocytes, basal prostatic cells, glandular and luminal cells, thymic epithelial cells, Leydig cells, extravillous trophoblasts, endothelial cells, smooth muscle cells, adipocytes, melanocytes, fibroblasts, peritubular cells, T-cells, NK-cells, monocytes, macrophages, Hofbauer cells, Kupffer cells, dendritic cells; and 3 cell types exhibit a proportion of upregulated CD marker genes within the cell-type markers that equal to or greater than 5%, including endometrial stromal cells, granulocytes, and Langerhans cells.

Taken together, these results have demonstrated that VSMCs are innate immune cells as judged by the upregulation of innate immune genes, cytokine and chemokine genes, plasma membrane protein genes and CD marker genes in VSMCs in response to various DAMPs, and the upregulation of plasma membrane and CD marker genes indicated the trans-differentiation potentials of VSMCs into 30 new cell types and 23 new cell types, respectively.

### Cholesterol, LPS, and IL-17 treatment in VSMCs were stronger stimuli than others for upregulating nuclear stress genes in the nuclear membrane, nucleoli, and nucleoplasm

**Figure 8:**
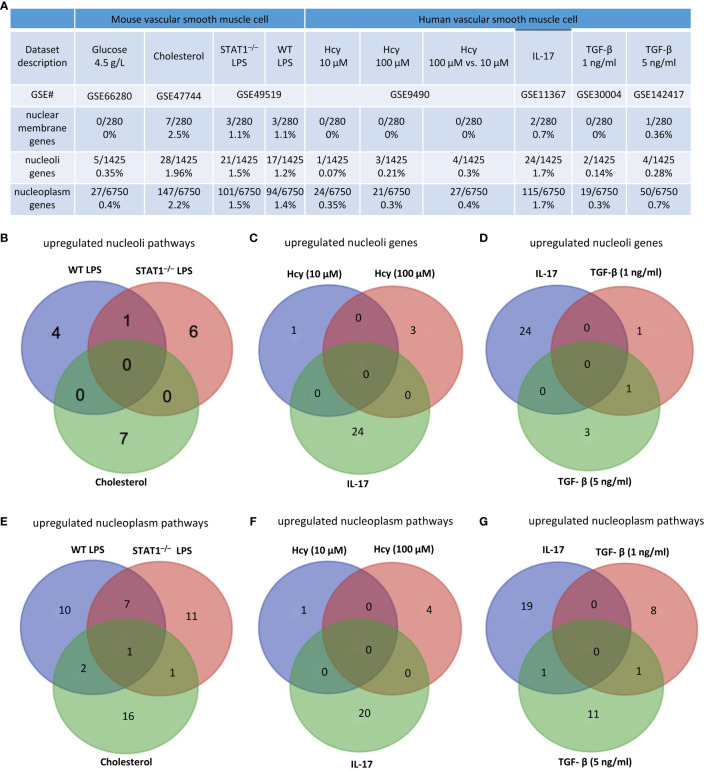
Nuclear membrane genes, nucleoli genes and nucleoplasm genes upregulation in mouse and human vascular smooth muscle cells (VSMCs) under atherogenic conditions such as high glucose, cholesterol, lipopolysaccharides (LPS), homocysteine (Hcy), interleukin-17 (IL-17), and transforming growth factor-B (TGF-B). **(A)** Nuclear membrane genes, nucleoli genes, and nucleoplasm genes were upregulated in mouse VSMCs and human VSMCs under atherosclerosis conditions. **(B)** 11 upregulated nucleoli pathways in LPS stimulated VSMCs were different from that of VSMCs stimulated by cholesterol loading. **(C)** 24 upregulated nucleoli genes in IL-17 stimulated VSMCs were different from that of VSMCs stimulated by homocysteine. **(D)** 24 upregulated nucleoli genes in IL-17 stimulated VSMCs were different from that of VSMCs stimulated by TGF-B. **(E)** 10 upregulated nucleoplasm pathways in LPS stimulated VSMCs were different from 16 upregulated nucleoplasm pathways in VSMCs stimulated by cholesterol loading. **(F)** 5 upregulated nucleoplasm pathways in homocysteine stimulated VSMCs were different from 20 upregulated nucleoplasm pathways in VSMCs stimulated by cholesterol loading. **(G)** 19 upregulated nucleoplasm pathways in IL-17 stimulated VSMCs were different from 20 upregulated nucleoplasm pathways in VSMCs stimulated by TGF-B. 280 nuclear membrane genes were collected from the nuclear membrane gene database in the HPA database (https://www.proteinatlas.org/humanproteome/subcellular/nuclear+membrane). 1425 nucleoplasm genes were collected from the nucleoli gene database in the HPA database (https://www.proteinatlas.org/humanproteome/subcellular/nucleoli). 6753 nucleoplasm genes were collected from the nucleoplasm gene database in the HPA database (https://www.proteinatlas.org/humanproteome/subcellular/nucleoplasm).

We hypothesized that, as innate immune cells, the VSMC nucleus responds to DAMP stimulations by upregulating genes in the nuclear membrane, nucleoli, and nucleoplasm. As shown in **Figure 8A**, 0%, 2.5%, 1.1%, and 1.1% of nuclear membrane protein genes were upregulated in VSMCs stimulated by high glucose, cholesterol, STAT1^–/–^ LPS, and WT LPS, respectively. Also, 0.35%, 1.96%, 1.5%, and 1.2% of nucleoli genes were upregulated in VSMCs stimulated by high glucose, cholesterol, STAT1^–/–^ LPS, and WT LPS, respectively. In addition, 0.4%, 2.2%, 1.5%, and 1.4% of nucleoplasm genes were upregulated in VSMCs stimulated by high glucose, cholesterol, STAT1^–/–^ LPS, and WT LPS, respectively. Moreover, 0%, 0%, 0%, 0.7%, 0%, and 0.36% of nuclear membrane protein genes were upregulated in human VSMCs stimulated by Hcy (10 μM), Hcy (100 μM), Hcy (100 μM) versus Hcy (10 μM), IL-17, TGF-β (1 ng/ml), and TGF- β (5 ng/ml), respectively. Furthermore, 0.07%, 0.21%, 0.3%, 1.7%, 0.14%, and 0.28% of nucleoli protein genes were upregulated in human VSMCs stimulated by Hcy (10 μM), Hcy (100 μM), Hcy (100 μM) versus Hcy (10 μM), IL-17, TGF- β (1 ng/ml), and TGF- β (5 ng/ml), respectively. Finally, 0.35%, 0.3%, 0.4%, 1.7%, 0.3%, and 0.7% of nucleoplasm protein genes were upregulated in human VSMCs stimulated by Hcy (10 μM), Hcy (100 μM), Hcy (100 μM) versus Hcy (10 μM), IL-17, TGF- β (1 ng/ml), and TGF- β (5 ng/ml), respectively. The upregulated nuclear membrane genes in VSMCs stimulated by LPS, IL-17, Hcy, cholesterol, and TGF-B were shown in the **Supplementary Figures S5A–D**.

We also noticed that upregulations of nucleoli genes or pathways in VSMCs were different under different stimuli. *i)* 4, 6, and 7 of upregulated pathways were specific to WT LPS-stimulated VSMCs, STAT1^–/–^ LPS-stimulated VSMCs, and cholesterol-loaded VSMCs, respectively (**Figure 8B**); *ii)* 1, 3, and 24 of upregulated nucleoli genes were specific to Hcy (10 μM)-treated VSMCs, Hcy (100 μM)-treated VSMCs, and IL-17-treated VSMCs, respectively (**Figure 8C**); and *iii)* 24, 1, and 3 of upregulated nucleoli genes were specific to IL-17-treated VSMCs, TGF- β (1 ng/ml)-treated VSMCs, and TGF- β (5 ng/ml)-treated VSMCs, respectively (**Figure 8D**). The upregulated nucleoli genes in VSMCs stimulated by LPS, IL-17, Hcy, cholesterol, and TGF-β were shown in the **Supplementary Figures S5E–H**. In addition, we also noticed that upregulated pathways of nucleoplasm genes in VSMCs were different under different stimuli: *a)* 10, 11, and 16 upregulated nucleoplasm pathways were specific to WT LPS-stimulated VSMC, STAT1^–/–^ LPS-stimulated VSMCs, and cholesterol-loaded VSMCs, respectively (**Figure 8E**); *b)* 1, 4, and 20 upregulated nucleoplasm pathways were specific to Hcy (10 μM)-treated VSMCs, Hcy (100 μM)-treated VSMCs, and IL-17-treated VSMCs (**Figure 8F**); and *c)* 19, 8, and 11 upregulated nucleoplasm pathways were specific to IL-17-treated VSMCs, TGF-β (1 ng/ml)-treated VSMCs, and TGF-β (5 ng/ml)-treated VSMCs (**Figure 8G**).

### Cholesterol, LPS, and IL-17 treatment in VSMCs were stronger stimuli than others for upregulating TFs, and upregulations of pioneer TFs are disease-stage-specific events in atherosclerosis and aortic aneurysms and are DAMP-specific events in VSMCs

**Figure 9:**
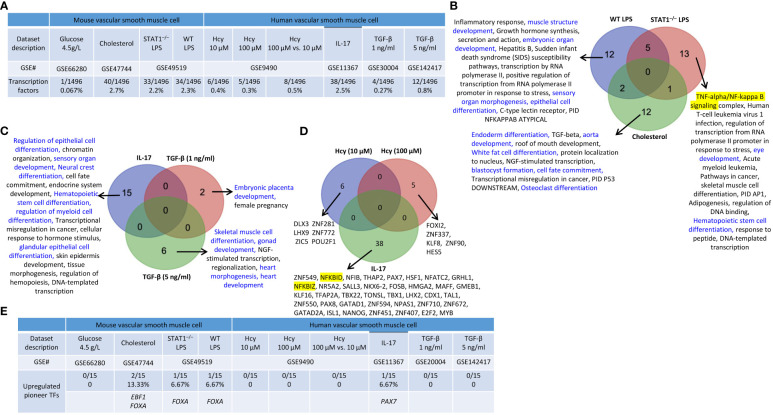
Transcription factor (TF) genes and pioneer TF genes upregulation in mouse and human vascular smooth muscle cells (VSMCs) stimulated by high glucose, cholesterol, lipopolysaccharides (LPS), homocysteine (Hcy), interleukin-17 (IL-17), and transforming growth factor-β (TGF-B). **(A)** TFs were significantly upregulated in mouse and human VSMCs under atherosclerosis conditions. **(B)** 12 upregulated TFs pathways were found upregulated in WT LPS VSMCs; 13 upregulated TFs pathways were found upregulated in STAT1^–/–^ LPS VSMCs; 12 upregulated TFs pathways were found specific in cholesterol loaded VSMCs. Differentiation pathways were shown in blue and master pathways were highlighted in yellow. **(C)** 15 upregulated TFs pathways were found specific in IL-17 treated VSMCs; 2 upregulated TFs were found specific in TGF-B (1 ng/ml) treated VSMCs; 6 upregulated TFs were found specific in TGF-B (5 ng/ml) treated VSMCs. Differentiation pathways were shown in blue. **(D)** 6 upregulated TF genes were found specific in Hcy (10 µM), 5 upregulated TF genes were found specific in Hcy (100 µM), and 38 upregulated TF genes were found specific in IL-17 treated VSMCs. Master genes were highlighted in yellow. **(E)** 3 pioneer TFs, including EBF1, FOXA, and PAX7, plays significant role in vascular smooth muscle cell pathologies. 15 pioneer TFs were collected from (PMID: 29507097).

We hypothesized that VSMCs, as innate immune cells, respond to DAMP stimulations by upregulating TF genes. As shown in **Figure 9A**, 0.067%, 2.7%, 2.2%, and 2.3% of TF genes were upregulated in VSMCs stimulated by high glucose, cholesterol, STAT1^–/–^ LPS and WT LPS, respectively. In addition, 0.4%, 0.3%, 0.5%, 2.5%, 0.27%, and 0.8% of TF genes were upregulated in human VSMCs stimulated by Hcy (10 μM), Hcy (100 μM), Hcy (100 μM) versus Hcy (10 μM), IL-17-treated VSMCs, and VSMCs stimulated by TGF-β (1 ng/ml) and TGF-β (5 ng/ml), respectively.

We also noticed that upregulations of TF genes or pathways in VSMCs were different under different stimuli: *i)* 12 of the upregulated TF pathways were specific to WT LPS-stimulated VSMCs which include 4 differentiation pathways (highlighted in blue) such as muscle structure development, embryonic organ development, sensory organ morphogenesis, and epithelial cell differentiation; 13 of the upregulated TF pathways were specific to STAT1^–/–^ LPS-stimulated VSMCs which include 2 differentiation pathways (highlighted in blue) such as eye development and Hematopoietic stem cell differentiation, and one master TF pathway NF-kappa B signaling; and 12 of the upregulated TF pathways were specific to cholesterol-loaded VSMCs including 6 differentiation pathways such as endoderm differentiation, aorta development, white fat cell differentiation, blastocyst formation, cell fate commitment, and osteoclast differentiation (**Figure 9B**); *ii)* 15 of the upregulated TF pathways were specific to IL-17-treated VSMCs including 6 differentiation pathways (highlighted in blue) such as regulation of epithelial cell differentiation, sensory organ development, neural crest differentiation, hematopoietic stem cell differentiation, regulation of myeloid cell differentiation, and glandular epithelial cell differentiation; 2 of the upregulated TF pathways were specific to TGF-β (1 ng/ml)-treated VSMCs including one differentiation pathway (highlighted in blue) embryonic placenta development; 6 of the upregulated TF pathways were specific to TGF-β (5 ng/ml)-treated VSMCs including skeletal muscle cell differentiation, gonad development, heart morphogenesis, and heart development (**Figure 9C**); and *iii)* 6, 5, and 38 upregulated TFs genes were specific to Hcy (10 μM)-treated VSMCs, Hcy (100 μM)-treated VSMCs, and IL-17-treated VSMCs (**Figure 9D**). The upregulated TFs in VSMCs under different DAMP stimulations were shown in the **Supplementary Figures S6A–F**.

In addition, we also found that three pioneer TFs, including *EBF1*, *FOXA*, and *PAX7* were upregulated in VSMCs stimulated by various DAMPs (**Figure 9E**). *EBF1* and *FOXA* were upregulated in cholesterol-loaded VSMCs, but *FOXA* was also upregulated in LPS-stimulated VSMCs. *PAX7* was upregulated in IL-17-stimulated VSMCs. Taken together, these results demonstrated that upregulation of pioneer TFs in VSMCs is DAMPs-stimulation-specific.

### Upregulated TFs only in nucleoli were decreased from 3.14% to 1.47% in aortic diseases and to 0% in VSMCs responding to various DAMPs; non-nucleus-localized TFs were decreased from 21.7% to 13.7% in aortic diseases and 18.05% in VSMC inflammation; and a few histone modification enzymes were upregulated and also localized in nucleoplasm

**Figure 10:**
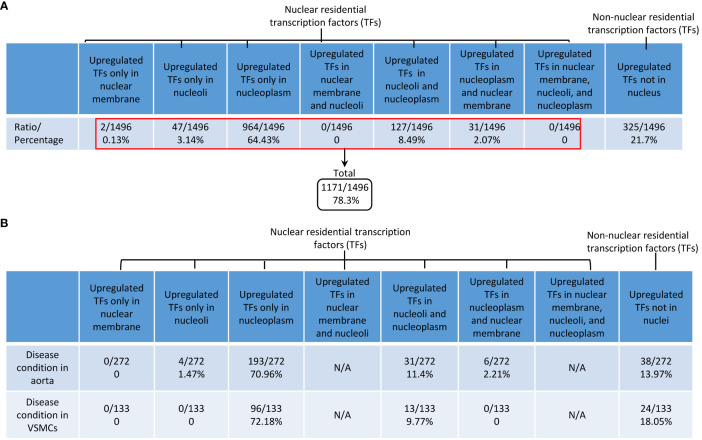
DAMPs-upregulated transcription factors are DAMPs-responsive transcription factors (TFs); and the majorities of DAMPs-responsive TFs were located in 3 locations, nucleoplasm TFs, nucleoli and nucleoplasm shared TFs, and non-nucleus residential TFs in aortic diseases and DAMPs-stimulated vascular smooth muscle cells. **(A)** Based on subcellular locations, all the human TFs were classified into 8 new subgroups: i) 0.13% nuclear member TFs; ii) 3.14% nucleoli TFs; iii) 64.43% nucleoplasm TFs; iv) 8.49% nucleoli and nucleoplasm shared TFs; v) 2.07% nucleoplasm and nuclear membrane shared TFs; and vi) 21.7% of non-nuclear residential TFs as cytosolic signal relaying TFs. Of note, two subgroups had no TFs. **(B)** TFs in nucleoplasm and TFs in nucleoli and nucleoplasm were increased in atherosclerosis, AAA and VSMC pathologies. In contrast, non-nucleus TFs were decreased in aortic diseases and VSMCs pathologies.

Previous reports classified TFs into basic helix-loop-helix TFs, zinc finger TFs, homeobox TFs, leucine zipper TFs, and nuclear receptor TFs based on their structural features (https://www.tutorialspoint.com/transcription-factors-definition-effects-and-types#) (97). We and others reported that TFs such as NF-kB (19) and AP-1 (8, 98) migrate from the cytosol to the nucleus in activated cardiovascular cells in response to DAMP stimulation. We hypothesized that transcription factor trafficking from the cytosol to the nucleus and sub-nuclear compartments is modulated in aortic diseases and VSMC inflammation. Based on subcellular localization, we used a Venn diagram (not shown due to space limits) and classified 1496 TFs into possible eight groups in physiological conditions, including *1)* 0.13% nuclear membrane TFs, *2)* 3.14% nucleoli TFs, *3)* 64.43% nucleoplasm TFs, *4)* 0% nuclear membrane and nucleoli TFs, *5)* 8.49% nucleoli and nucleoplasm TFs, *6)* 2.07% nuclear membrane and nucleoplasm TFs, *7)* 0% nuclear membrane, nucleoplasm, and nucleoli TFs, and *8)* 21.7% non-nucleus TFs as cytosolic signal relaying TFs (**Figure 10A**). As shown in **Figure 10B**, *i)* upregulated nucleoli TFs were decreased from 3.14% to 1.47% in aortic diseases, and 0% in VSMCs responding to various DAMPs, which was in a contrast to the roles of nucleoli as a nuclear stress organelle; *ii)* upregulated nucleoplasm TFs were increased from 64.43% to 70.96% in aortic diseases and 72.18% in VSMCs in response to DAMPs; *iii)* upregulated nucleoli and nucleoplasm TFs were increased from 8.49% to 11.3% in aortic diseases and 9.77% in VSMCs responding to DAMPs; and *iv)* non-nucleus-localized TFs were decreased from 21.7% to 13.7% in aortic diseases and 18.05% in VSMC inflammation, which was a contrast to the posttranslational activation and migration from cytosol to nucleus of well-characterized TFs such as NF-kB and AP-1. Our results have demonstrated for the first time that the nuclear and cytosol localizations of TFs are changed in aortic diseases and VSMC immunity, which are new additions to the current TF classification and functions.

**Figure 11:**
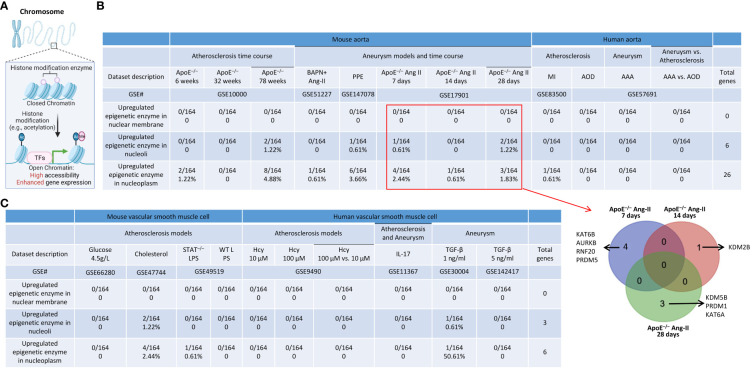
A few upregulated histone modification enzymes in aortic diseases and VSMCs stimulated by DAMPs were localized in nucleoli and nucleoplasm. **(A)** Schematic diagram showed that histone modification enzymes facilitate the modification process (e.g., acetylation), enabling the binding of TFs to specific DNA sequences and enhance gene expression. **(B)** A total of 10 histone modification enzymes were upregulated in ApoE^–/–^ HFD feeding for 78 weeks; A total of 7 histone modification enzymes were upregulated in PPE induced AAA; A total of 7 histone modification enzymes were upregulated in ApoE^–/–^ Ang II 7 days, 14 days (1 enzyme) and 28 days (5 enzymes), respectively. Four upregulated histone modification enzymes in the aorta of ApoE^–/–^ Ang II AAA for 7 days were different from that of one upregulated enzyme in Ang II AAA for 14 days and that of Ang II AAA for 28 days and no overlaps were found among three time points. **(C)** A few upregulated histone modification enzymes in VSMCs stimulated by various DAMPs were localized in nucleoli and nucleoplasm.

As we reported, epigenetic memory plays significant roles in establishing trained immunity and inflammation (4, 67, 98), and the expressions of the majority of histone modification enzymes in CVDs and other inflammations are downregulated (99). Histone modification enzymes facilitate the modification process (e.g., acetylation), enabling the binding of TFs to specific DNA sequences and enhancing gene expression (**Figure 11A**). We hypothesized that the expressions of a minority of histone modification enzymes are upregulated in aortic diseases and VSMC inflammation. As we reported, a few histone modification enzymes were upregulated in ApoE^–/–^ at 6 weeks, 78 weeks, PPE-AAA, ApoE^–/–^ Ang II-AAA at 7 days and 28 days, cholesterol-loaded VSMCs, and TGF-β (1 ng/ml)-stimulated VSMCs, and the rest of aortic diseases and VSMC inflammations had no upregulation of histone modification enzymes (**Figures 11B, C**). The majority of upregulated enzymes were localized in nucleoli and nucleoplasm, which was similar to that of upregulated TFs. The 8 upregulated enzymes in ApoE^–/–^ Ang II-AAA in three time points, such as 7 days, 14 days, and 28 days, were not overlapped (**Figure 11B**).

### Atherosclerosis was associated with upregulation of oxidative, necrotic, and pyroptotic cell death genes and efferocytosis genes; PPE-AAA upregulated more cell death genes than that of other AAA models; ApoE^–/–^ Ang II-AAA at 7 days and 28 days upregulated more cell death genes than that of 14 days; and cholesterol loading upregulated more oxidative death genes in VSMCs, whereas LPS induced more necrotic death genes in VSMCs

**Figure 12:**
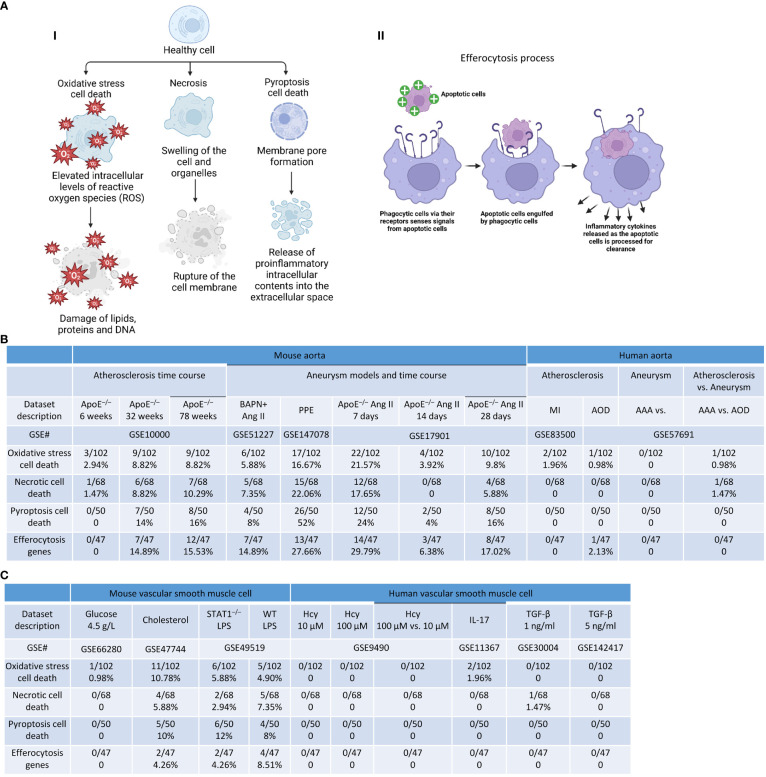
Cell death genes and efferocytosis were upregulated in aortic diseases and VSMCs stimulated by DAMPs. **(A)** (i) Schematic diagram showed the three types of cell death and their aspects, (i) Schematic diagram demonstrated efferocytosis process. **(B)** Cell death genes and efferocytosis genes were significantly upregulated in atherosclerosis and aortic aneurysm. **(C)** Cell death genes and efferocytosis genes were significantly upregulated vascular smooth muscle cells stimulated with atherogenic stimuli. Oxidative stress cell death genes were collected from Mouse Genome Informatics (http://www.informatics.jax.org/go/term/GO:0070269). Necrotic cell death genes were collected from Mouse Genome Informatics (http://www.informatics.jax.org/go/term/GO:0070265), Pyroptosis cell death genes were collected form Mouse Genome Informatics (https://www.informatics.jax.org/go/term/GO:0070269), Efferocytosis genes were collected from Mouse Genome Informatics (https://www.informatics.jax.org/vocab/gene_ontology/GO:0043277).

We previously reported that cell death plays significant roles in activated T cells (20, 56, 100–102), CD4^+^Foxp3^+^ Tregs in vascular inflammation (18, 19), activated ECs in atherosclerosis (8, 11, 33, 103), MI (104), angiogenesis (105), activated VSMCs in neointima hyperplasia (12), non-alcoholic fatty liver disease (106), ischemia-reperfusion in the liver (107), and ultrasound-treated cancer cells (57, 108, 109). We hypothesized that oxidative stress cell death, necrotic cell death (110), and pyroptotic cell death play significant roles in atherosclerosis, aortic aneurysms, and VSMC inflammation (37, 111–114) (**Figure 12A**). As shown in **Figure 12B**, we found that: *1)* 2.9%, 8.8%, and 8.8% of oxidative stress-induced cell death genes; 1.47%, 8.8%, and 10.29% of necrotic cell death genes; 0%, 14%, and 16% of pyroptosis cell death were upregulated in ApoE^–/–^ at 6 weeks, 32 weeks, and 78 weeks, respectively; *2)* 5.88% and 16.67% of oxidative stress cell death genes; 7.35% and 22.06% of necrotic cell death genes; 8% and 52% of pyroptosis cell death were upregulated in BAPN+Ang II-AAA and PPE-AAA, respectively; *3)* 21.57%, 3.92%, and 9.8% of oxidative stress cell death genes; 17.65%, 0%, and 5.88% of necrotic cell death genes; and 24%, 4%, and 16% of pyroptosis cell death were upregulated in ApoE^–/–^ Ang II-AAA at 7 days, 14 days, and 28 days, respectively, also indicating the twin peak pattern as found in innate immune genes and nuclear stress genes.

On the other hand, the removal of billions of apoptotic cells from the system via the process of efferocytosis is essential for homeostasis, which is actively anti-inflammatory, promotes resolution, and prevents aneurysm (96). However, aberrations in efferocytosis are associated with numerous inflammatory pathologies, including atherosclerosis (97) and probably aortic aneurysm (**Figure 12A**). We hypothesized that efferocytosis genes are upregulated in atherogenesis and aortic aneurysm models. As shown in **Figure 12B**, 0%, 14.89%, and 15.53% of efferocytosis genes were upregulated in ApoE^–/–^ at 6 weeks, 32 weeks, and 78 weeks, respectively; PPE-AAA upregulated more efferocytosis genes (27.66%) than that of other AAA models; ApoE^–/–^ Ang II-AAA at 7 days and 28 days upregulated more efferocytosis genes (29.79% and 17.02%) than that of 14 days. These results demonstrated that upregulation of efferocytosis genes may play important roles in atherosclerosis and aortic aneurysms. We also noticed that upregulations of cell death genes in VSMCs were different under different stimuli. *i)* 0.98%, 10.78%, 5.88%, and 4.9% of oxidative stress cell death genes, 0%, 5.88%, 2.94%, and 7.35% of necrotic cell death genes, 0%, 10%, 12%, and 8% of pyroptosis cell death were upregulated in VSMCs stimulated by high glucose, cholesterol, STAT1^–/–^ LPS and WT LPS, respectively; *ii)* 0%, 0%, 1.96%, 0%, and 0% of oxidative stress cell death genes, 0%, 0%, 0%, 1.47%, and 0% of necrotic cell death genes were upregulated in Hcy (10 μM)-treated VSMCs, Hcy (100 μM)-treated VSMCs, IL-17-treated VSMCs, TGF-β (1 ng/ml)-treated VSMCs, and TGF-β (5 ng/ml)-treated VSMCs, respectively (**Figure 12C**). We also examined the efferocytosis in VSMCs treated with different stimulations. As shown in **Figure 12C**, 0%, 4.26%, 4.26%, and 8.51% of efferocytosis genes were upregulated by high glucose, cholesterol, STAT1^–/–^ LPS and WT LPS, respectively.

### Several fibrosis genes, VSMC trans-differentiation genes, and other cell type trans-differentiation genes were upregulated in atherosclerosis and aortic aneurysm diseases

**Figure 13:**
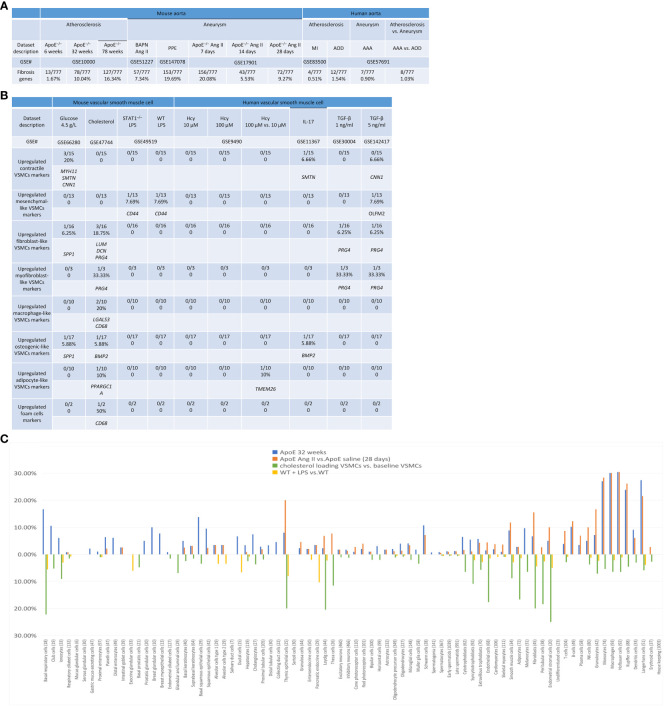
Fibrosis genes, trans-differentiation VSMCs markers, and cell type-specific markers of some human 79 cell types were upregulated in atherosclerosis and abdominal aortic aneurysm (AAA) diseases as well as cholesterol-loaded and LPS stimulated VSNCs. **(A)** Fibrosis genes were upregulated in mouse and human aortic diseases. **(B)** Cholesterol loading upregulated marker genes of fibroblasts, macrophages, osterogenic, adipocyte-like cells; and foam cells; LPS upregulated TGF-B upregulated markers of mesenchymal cells; homocysteine upregulated adipocyte marker; IL-17 upregulated osteogenic cell marker; and TGF-B upregulated markers of fibroblast and myofibroblast. (Total 82 trans-differentiation markers in seven cell types). **(C)** Cell type-specific markers of some human 79 cell types identified in the Human Protein Atlas were upregulated in ApoE^–/–^ mouse aorta fed with HFD for 32 weeks, Ang II infused ApoE^–/–^ AAA aorta, and VSMCs stimulated by cholesterol and LPS, indicating trans-differentiation of residential cells in aorta and VSMCs and inflammatory cell migration from circulating blood into aorta. Fibrosis genes were collected from (PMID: 33519923).

Fibrosis is the compensating process for tissue injury caused by chronic inflammation, which is initially beneficial and maintains extracellular homeostasis. However, in the case of atherosclerosis and aortic aneurysms, activated resident fibroblasts and VSMCs perpetually remodel the extracellular matrix (ECM) under the control of autocrine and paracrine signaling from the immune cells. In consequence, excessive ECM secretion, disorganized ECM, and thickening of the affected tissue result (115). We hypothesized that fibrosis genes are upregulated in the atherogenesis and aortic aneurysm models. As shown in **Figure 13A**, 1.67%, 10%, and 16.34% of fibrosis genes were upregulated in ApoE^–/–^ at 6 weeks, 32 weeks, and 78 weeks, respectively; PPE-AAA upregulated more fibrosis genes (19.69%) than that of other AAA models; ApoE^–/–^ Ang II-AAA at 7 days and 28 days upregulated more fibrosis genes (20% and 9.27%) than that of 14 days. These results demonstrated that upregulation of fibrosis genes may play important roles in atherosclerosis and aortic aneurysms.

Previous reports showed that VSMCs in CVDs can be transdifferentiated into other seven cell types, including 82 markers from mesenchymal stem cell-like VSMCs (48), fibroblast-like VSMCs, myofibroblast-like VSMCs (48), macrophage-like VSMCs, foam cell-like VSMCs (48), osteogenic-like VSMCs (48), and adipocyte-like VSMCs (46–48). We hypothesized that the expression of these seven cell-type marker genes is upregulated in VSMCs stimulated by various DAMPs.

As shown in **Figure 13B**, *PRG4* was the most frequently upregulated fibroblast-like and myofibroblast-like gene in VSMCs stimulated by cholesterol and TGF-β; *SPP1* was the upregulated fibroblast and osteogenic gene in VSMCs stimulated by high glucose; *CNN1* was the upregulated contractile gene marker in VSMCs stimulated by high glucose and TGF-β; *MYA11* and *SMTN* were the upregulated contractile gene markers in VSMCs stimulated by high glucose and IL-17; *CD44* and *OLFM2* were the mesenchymal-like marker upregulated in VSMCs stimulated by LPS and TGF-β; *LGALS3* and *CD68* were the upregulated macrophage-like and foam cell markers in VSMCs stimulated by cholesterol; *BMP2* was the upregulated osteogenic marker in VSMCs simulated by cholesterol and IL-17; and *PPARGC1* and *TMEM26* were the upregulated adipocyte markers in VSMCs stimulated by cholesterol and Hcy. Taken together, these results have demonstrated that cholesterol and TGF-β upregulate multiple cell type markers in VSMC, and high glucose, high Hcy, LPS, and IL-17 upregulate one or two cell type markers, suggesting that trans-differentiation of VSMCs to multiple cell types is induced under those stimuli.

It has been documented that seven cell types were identified in cell trans-differentiation. However, a significant question remained whether cell trans-differentiation in atherosclerosis or AAA is only limited to the seven documented cell type trans-differentiation or can be potentially trans-differentiated to other cell types. Of note, the NIH-funded human protein atlas database explores all the cell type specific marker expressions, which provide a new opportunity, and a unique and non-biased manner to examine the trans-differentiation potential with the markers of all the cell types identified so far. Therefore, we used this approach to examine the trans-differentiation potentials in the mouse aortas with atherosclerosis and AAA. We collected 8065 genes for 79 cell type markers from the “very high” gene groups of all the tissue cell type sections in the HPA (https://www.proteinatlas.org/humanproteome/tissue+cell+type) in order to determine whether VSMCs increase the expression of some of those 79 cell type markers in response to DAMPs. As shown in **Figure 13C**, since 79 cell types have different numbers of very high gene numbers as markers, the percentage was presented by dividing the numbers of upregulated gene markers by the total number of markers for any given specific cell type. Four groups were selected to be representatives from all the aortic disease groups and VSMC groups, including ApoE^–/–^ at 32 weeks, ApoE^–/–^ Ang II at 28 days, cholesterol-loaded VSMCs, and WT LPS-stimulated VSMCs, to be analyzed. The results showed that: *1)* the 31 cell types exhibiting a proportional increase of 5% or more cell type markers in ApoE^–/–^ at 32 weeks included basal respiratory cells, club cells (nonciliated secretory epithelial cells present in bronchioles of distal pulmonary airways) (116), breast glandular cells, basal squamous epithelial cells, Schwann cells (required for successful nerve regeneration; they partially “de-differentiate” in response to injury, re-initiating the expression of developmental genes that support nerve repair), B-cells, monocytes, macrophages, Hofbauer cells [placental villous macrophages of fetal origin that are present throughout pregnancy (117)], Kupffer cells (resident liver macrophages and play a critical role in maintaining liver functions) (118), Langerhans cells (originate from the bone marrow and then migrate into the epithelium to perform the function of antigen recognition and presentation) (119), ionocytes, paneth cells, distal enterocytes, prostatic glandular cells, breast myoepithelial cells, basal keratinocytes, squamous epithelial cells, ductal cells, cholangiocytes, thymic epithelial cells, cytotrophoblasts, syncytiotrophoblasts, extravillous trophoblasts, smooth muscle cells, melanocytes, fibroblasts, endometrial stromal cells, NK-cells, granulocytes, and dendritic cells; *2)* the 18 cell types exhibiting a proportional increase of 5% or more cell type markers in ApoE^–/–^ Ang II-AAA at 28 days included thymic epithelial cells, Leydig cells, theca cells, Schwann cells, smooth muscle cells, fibroblasts, endometrial stromal cells, T-cells, B-cells, plasma cells, NK-cells, granulocytes, monocytes, macrophages, Hofbauer cells, Kupffer cells, dendritic cells, and Langerhans cells; *3)* the 18 cell types exhibiting a proportional increase of or more cell type markers in cholesterol-loaded VSMCs, exclude the trans-differentiation cell types discussed earlier, include basal respiratory cells, thymic epithelial cells, Leydig cells (produce the high levels of androgen, testosterone or androstenedione, required for differentiation of male genitalia and brain masculinization) (120), theca cells (differentiate into small luteal cells that produce progesterone in response to luteinizing hormone) (121), syncytiotrophoblasts [a continuous, specialized layer of placenta epithelial cells. They cover the entire surface of villous trees and are in direct contact with maternal blood (https://www.ncbi.nlm.nih.gov/books/NBK53245/)], endothelial cells, peritubular cells, endometrial stromal cells, club cells, ionocytes, glandular and luminal cells, cytotrophoblasts, extravillous trophoblasts, melanocytes, granulocytes, monocytes, Hofbauer cells, and Langerhans cells; and *4)* 6 cell types exhibiting a proportional increase of 5% or more in LPS-stimulated VSMCs, exclude the trans-differentiation cell types discussed earlier, include basal respiratory cells, exocrine glandular cells, ductal cells, thymic epithelial cells, pancreatic endocrine cells, and endometrial stromal cells.

Taken together, these results have demonstrated for the first time that *1)* atherosclerotic aorta and AAA aorta, *i)* increase inflammatory cell type recruitment into aorta, *ii)* upregulate a few other structural cell types for trans-differentiation of vascular cells and immune cells, and *iii)* increase proliferation of new cell types in aortic diseases; and *2)* upregulation of 18 new cell type markers in cholesterol-loaded VSMCs and upregulation of 6 new cell types in LPS-stimulated VSMCs suggest new trans-differentiation potentials of VSMCs stimulated by DAMPs.

### Upregulated innate immune and inflammatory genes from 12 groups were upregulated in antioxidant transcription factor NRF2^–/–^ transcriptomic datasets and downregulated in other 5 master gene-deficient transcriptomic datasets, including AHR^–/–^, NF-kB^–/–^ with and without LPS stimulation, ROS generating enzyme NOX2^–/–^, PERK^–/–^ with and without tunicamycin stimulation, and SET7^–/–^

**Figure 14:**
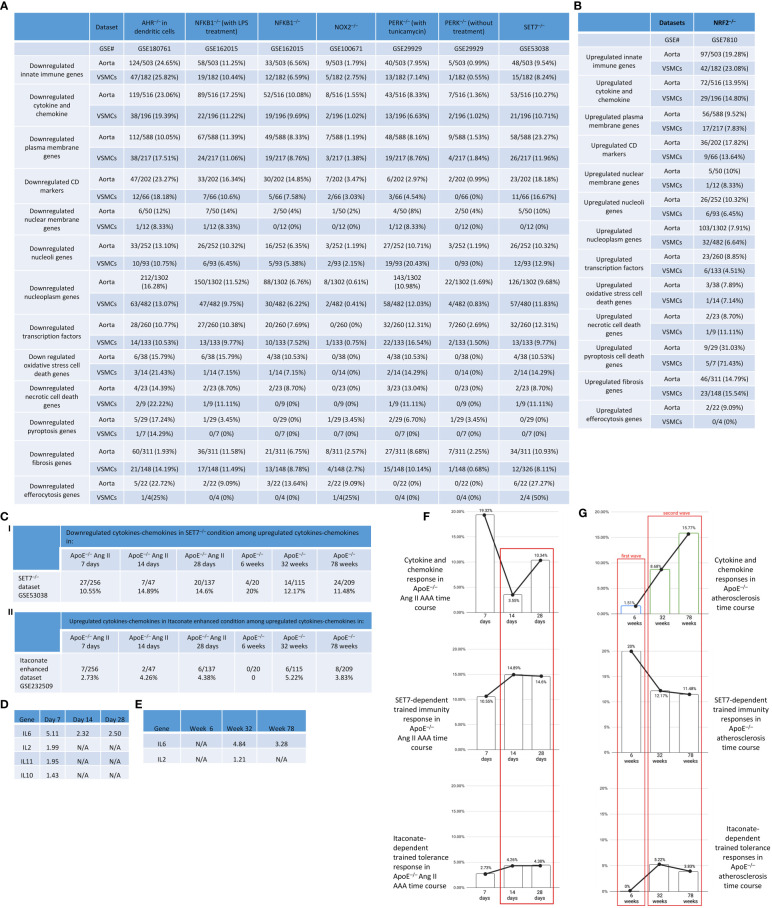
Some of upregulated innate immune genes and other 11 groups of related upregulated genes were promoted by AHR, NF-kB, NOX2, PERK and SET7 but were suppressed by antioxidant transcription factor NRF2. **(A)** Some of upregulated innate immune genes and other 11 groups of related upregulated genes were downregulated in the transcriptomic datasets of AHR^–/–^, NF-KB^–/–^ with LPS, NF-kB^–/–^ without treatment, NOX2^–/–^, PERK^–/–^ with tunicamycin treatment, PERK^–/–^ without treatment, SET7^–/–^. **(B)** Some of upregulated innate immune genes and other 11 groups of related upregulated genes were upregulated in NRF2^–/–^ transcriptomic dataset. **(C)** (i) Downregulated cytokines and chemokines in SET7^–/–^ condition among upregulated cytokines and chemokines in atherosclerotic and aneurysm aortic diseases. (ii) Upregulated cytokines and chemokines in Itaconate enhanced condition among upregulated cytokines and chemokines in atherosclerotic and aneurysm aortic diseases. **(D)** Anti-inflammatory cytokines such as IL6, IL2, Il11, and IL10 were upregulated in AAA. **(E)** Anti-inflammatory cytokines such as IL6 and IL2 were upregulated in atherosclerosis at 32 and 78 weeks of HFD feeding. **(F)** Cytokine and chemokine responses, SET7-dependent trained immunity responses, and itaconate-dependent trained tolerance response in ApoE^–/–^ Ang II-AAA time course. **(G)** Cytokine and chemokine responses, SET7-dependent trained immunity responses, and itaconate-dependent trained tolerance responses in ApoE^–/–^ atherosclerosis time course.

To elucidate the molecular mechanisms underlying upregulation of innate immune genes, cytokine and chemokine genes in aortic diseases and VSMC stimulated by DAMPs, we hypothesized that a list of master TFs and master genes are partially responsible for upregulation of those innate immune-related gene lists. As we have shown previously (**Figure 4E**), in the pathways upregulated in ApoE^–/–^ at 32 weeks, the aryl hydrocarbon receptor (AHR) pathway was upregulated. Also, in ApoE^–/–^ Ang II at 7 days, ApoE^–/–^ at 78 weeks, and VSMCs stimulated with LPS, the NF-kB pathway was upregulated (**Figures 2E**, **4C, D**). In IL-17-treated VSMCs, NFKBID and NFKBIZ were upregulated (**Figure 9D**). As shown in **Figures 14A, 14B**, upregulated genes from innate immune genes, cytokines and chemokines, plasma membrane proteins, nuclear membrane proteins, nucleoli proteins, nucleoplasm proteins, TFs, oxidative stress cell death genes, necrotic cell death genes, pyroptosis cell death genes, efferocytosis genes, and fibrosis genes were downregulated in 5 master gene-deficient transcriptomic datasets including inflammatory TFs AHR^–/–^ dataset (62, 122–124), nuclear factor-kB (NF-kB)^–/–^ with and without LPS stimulation dataset (125–127), superoxide-generating NADPH oxidase heavy chain subunit (NOX2)^–/–^ dataset (23, 37, 128, 129), endoplasmic reticulum (ER) stress (130) inducer) stimulation dataset, eukaryotic translation initiation factor 2 alpha kinase 3 (PRKR-like endoplasmic reticulum kinase, PERK^–/–^ (7, 131)) and PERK^–/–^ with tunicamycin (inhibitor of the UDP-N-acetylglucosamine-dolichol phosphate N-acetylglucosamine-1-phosphate transferase) dataset, trained immunity (innate immune memory)-promoting histone lysine methyltransferase SET7^–/–^ dataset (132, 133) and were upregulated in antioxidant TF NRF2^–/–^ transcriptomic datasets (11), respectively. Our analysis showed that AhR and PERK deficiency had the most significant downregulation of innate immune genes, cytokines and chemokines, oxidative stress cell death genes, and nuclear stress genes (**Figure 14A**).

Oxidative stress is one of the pivotal factors in the pathogenesis of atherosclerosis and AAA. NRF2 is crucial for the oxidative stress response, and the development of atherosclerosis and AAA is intimately linked to the NRF2 signaling pathway. NRF2 signaling controls a variety of physiological and pathological processes that are involved in the development of atherosclerosis, including inflammation, foam cell production, lipid homeostasis regulation, macrophage polarization, and redox regulation (134, 135). NRF2 deficiency in mice accelerates atherosclerotic lesions and increased expression of inflammatory cytokines, such as TNFα, IL-6 and IL-1β (136) as well as AAA formation (137). Therefore, we used the NRF2^–/–^ transcriptomic datasets to examine the upregulation of upregulated innate immune genes, cytokines and chemokines, and other gene lists in aortic diseases and VSMCs under different stimulation. Our analysis showed that NRF2 deficiency increased the upregulation of innate immune genes, cytokine and chemokine genes, CD marker genes, pyroptosis cell death genes, and fibrosis genes in aortic diseases and DAMPs stimulated VSMCs (**Figure 14B**). Furthermore, as shown in **Figure 14C**, among the 256 upregulated cytokines and chemokines in ApoE^–/–^ Ang II at 7 days, there were 27 and 7 cytokines and chemokines downregulated by SET7^–/–^ and itaconate, respectively; in 47 upregulated cytokines and chemokines in ApoE^–/–^ Ang II at 14 days, there were 7 and 2 cytokines and chemokines downregulated by SET7^–/–^ and itaconate, respectively; in 137 upregulated cytokines and chemokines in ApoE^–/–^ Ang II at 28 days, there were 20 and 6 cytokines and chemokines downregulated by SET7^–/–^ and itaconate, respectively; in 20 upregulated cytokines and chemokines in ApoE^–/–^ at 6 weeks, there were 4 and 0 cytokines and chemokines downregulated by SET7^–/–^ and itaconate, respectively; in 115 upregulated cytokines and chemokines in ApoE^–/–^ at 32 weeks, there were 14 and 6 cytokines and chemokines downregulated by SET7^–/–^ and itaconate, respectively; in 209 upregulated cytokines and chemokines in ApoE^–/–^ at 78 weeks, there were 24 and 8 cytokines and chemokines downregulated by SET7^–/–^ and itaconate, respectively. The anti-inflammatory cytokines that were upregulated in AAA at 7, 14, and 28 days include IL-6, Il-2, IL-11, and IL-10 (**Figure 14D**); however, the anti-inflammatory cytokines that were upregulated in atherosclerosis include IL-6 and IL-2 (**Figure 14E**).

Taken together, our results have demonstrated that TFs AHR and NF-kB, ER stress-promoting PERK, trained immunity (innate immune memory)-promoting SET7, and reactive oxygen species (ROS)-oxidative stress-generating enzyme NOX2 (37), and antioxidative TF NRF2 play significant roles in regulating upregulation of innate immune genes and other inflammatory genes in aortic diseases and VSMCs responding to DAMPs.

## Discussion

Regardless of the important progress made in the field, the following significant issues remain unaddressed: first, global innate immune responses in different stages of atherosclerosis and aortic aneurysms; second, are VSMCs innate immune cells; and third, transcriptomic reprogramming of all the nucleus-localized proteins responds to atherosclerosis and aortic aneurysms. To address these questions, we performed transcriptome analyses on ApoE^–/–^ Ang II abdominal aortic aneurysm (AAA), ApoE^–/–^ atherosclerosis time course, and AAA time course and made significant findings: *1)* 95% and 45% upregulated innate immune pathways (UIIPs, based on data of 1226 innate immune genes) in ApoE^–/–^ Ang II AAA at 7 days were different from that of 14 days and that of 28 days, respectively; and AAA showed twin peaks of UIIPs with a major peak at 7 days and a minor peak at 28 days; *2)* all the UIIPs in ApoE^–/–^ atherosclerosis at 6 weeks were different from that of 32 weeks and that of 78 weeks (two waves); *3)* analyses of additional 1325 cytokine/chemokine genes confirmed two-wave inflammation in atherosclerosis and twin-peak inflammation in AAA; *4)* DAMPs-stimulated VSMCs were innate immune cells as judged by the upregulation of innate immune genes and genes from 12 additional lists; *5)* DAMPs-stimulated VSMCs increased trans-differentiation potential by upregulating not only some of 82 markers of 7 VSMC-plastic cell types, but also 21 new cell types (out of 79 human cell types with 8065 cell markers); and *6)* analyses of gene deficient transcriptomes indicated that inflammatory TFs AhR, NF-kB, ROS enzyme NOX2, ER stress kinase PERK, and trained immunity-promoting histone methyltransferase SET7 promote but antioxidant TF NRF2 suppress 12 lists of innate immune gene upregulation in atherosclerosis, AAA, and DAMPs-stimulated VSMCs. Our findings have provided novel insights on the roles of innate immunity in aortic diseases and VSMC innate immunity.

ApoE^–/–^ mice on high fat diet for 6 weeks were the earliest time examined and showed monocyte adhesion to the endothelium and sporadic foam cells in the subendothelial space. In older mice around 32 weeks of HFD, the fibrous plaques were developed and showed a large necrotic core and abundant fibrous tissue, therefore this time point used to study vascular calcification. However, an elderly mice showed markedly accelerated atherogenesis. The pathogenesis of lesions in the apoE-deficient mice is remarkably similar to our understanding of atherogenesis in humans. Given that it shares several characteristics with human AAA acquisition, such as predominance of male gender in the setting of mild hypertension and increased incidence in the presence of hyperlipidemia, the ang II-induced mouse model in ApoE−/− mouse is a widely used rodent model that has been thoroughly examined. This model has a well-defined sequence of events that begins with early inflammatory macrophage infiltration into the medial layer of the aneurysm-prone area, which is rich in smooth muscle. Within the first seven days of AngII infusion, transmedial dissection causes rapid luminal expansion. Subsequently, complex inflammatory events occur, including the formation of intramural thrombus, the breakdown of elastin, and profound remodeling, in which the thrombus is frequently resorbed and replaced by fibrous tissue interspersed with leukocytes (138). The time frame of 28 days after Ang II infusion and high fat feeding is the most common duration were used to study Ang II-induced AAA. We reported that the secretomic signaling pathways in ApoE^–/–^ atherosclerosis at 6 weeks, 32 weeks and 78 weeks (78) are different; and the secretomic signaling pathways in ApoE^–/–^ Ang II at 7 days, 14 days and 28 days (79) are also different (22); and Treg-derived anti-inflammatory/immunosuppressive cytokine IL-35 inhibits inflammatory genes at the early phase and promotes tissue repair at the late phase of hind-limb ischemia (139). Others reported that inflammation occurs at the peak in 4 days after myocardial infarction, followed by inflammation resolution and repair (140) and that neutrophil extracellular traps (NETs, citrullinated histone 3, Cit-H3 as the readout) reach the peak at 3 days in ApoE^–/–^ Ang II-AAA (141, 142). However, the novel findings presented here demonstrated for the first time in atherosclerosis that although innate immune gene upregulation is increased from 6 weeks, 32 weeks to 78 weeks, the first wave of innate immune pathways in atherosclerosis at 6 weeks is switched off, and the second wave of innate immune pathways are turned on from 32 weeks and continued to 78 weeks. Although innate immune pathways are partially overlapped in ApoE^–/–^ Ang II at 7 days and 28 days, upregulation scales and signaling pathways of innate immune genes in ApoE^–/–^ Ang II at 7 days, 14 days and 28 days are different and show twin peaks, with the major peak at 7 days and the minor peak at 28 days. The time-course differences in innate immune responses in atherosclerosis and aortic aneurysms have provided novel insights into the development of new therapeutics. In ApoE^–/–^ Ang II-AAA at 14 days, upregulated cytokine and chemokine responses were decreased compared to those at 7 days. In contrast, trained immunity and trained tolerance levels increased at 14 days and stayed steady for 28 days. The results have demonstrated that the second peak of upregulated cytokines and chemokines in ApoE^–/–^ Ang II-AAA results from the interplay of proinflammatory SET7-dependent trained immunity and anti-inflammatory itaconate-dependent trained tolerance (**Figure 14F**). In the ApoE^–/–^ HFD atherosclerosis model, the initial 6-week innate immune pathways (first wave) vanished, giving way to a distinct set of innate immune pathways at 32 weeks, which continued at 78 weeks (second wave). However, SET7-dependent trained immunity (innate immune memory) and itaconate-dependent trained tolerance maintained a consistent level throughout the ApoE^–/–^ HFD atherosclerosis time course, rather than being diminished by the disappearance at 6 weeks innate immune pathway. This implies that the sustained increase in innate immune response during the second wave is a result of the interplay between trained immunity and trained tolerance (**Figure 14G**).

**Figure 15:**
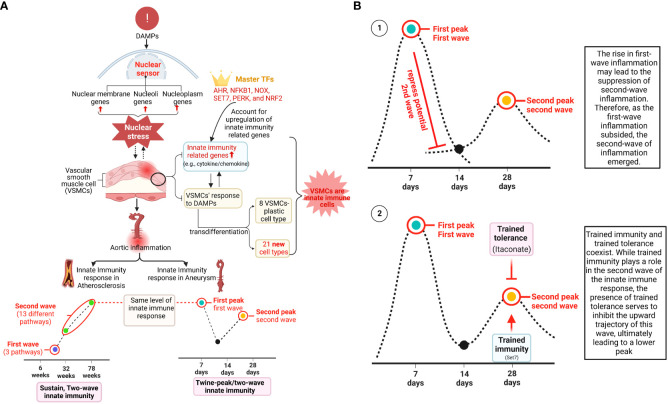
A new working model: **(A)** 1) We have identified atherosclerosis stage specific innate immune programs and abdominal aortic aneurysm (AAA) stage-specific innate immune programs (twin peak patterns); 2) We have also demonstrated that vascular smooth muscle cells are innate immune cells by upregulating innate immune genes and other 11 innate immune related groups of genes; 3) Atherosclerosis and AAA induce trans-differentiation of many new aortic cell types; 4) DAMPs induce vascular smooth muscle cell trans-differentiation into many new cell types; 5) Upregulation of nuclear residential protein genes in response to aortic diseases and DAMPs in VSMCs serves as a new integrated nuclear danger signal sensing program; 6) Oxidative stress cell death genes, necrotic cell death genes, and pyroptosis genes are upregulated in atherosclerotic aorta, AAA aorta and VSMCs stimulated by DAMPs. **(B)** i) The second wave of innate immune response peak identified in ApoE^–/–^ Ang-II AAA at 28 days is suppressed by the innate immune first peak; ii) interplay between SET7 promoted trained immunity (innate immune memory) and itaconate promoted trained tolerance may contribute to the second peak identified in ApoE^–/–^ Ang-II AAA at 28 days.

Based on our results, we propose a new working model (**Figure 15**). The innate immune responses are continuously on the rise from HFD at 6 weeks, 32 weeks to 78 weeks but innate immune pathways have the feature of two waves, with the first wave at 6 weeks and then switching to the second wave. Similarly, the innate immune responses in ApoE^–/–^ Ang II-AAA show a twin-peak pattern, with the first peak at 7 days and the second peak at 28 days. Our previous reports suggest that the first wave of inflammation may be different from the second wave and may also suppress the second wave of inflammation. The innate immune gene upregulations are not unique to ApoE^–/–^ Ang II-AAA model but are also found in other AAA mouse models with the highest innate immune responses identified in PPE-AAA model. VSMCs are innate immune cells as judged by the upregulation of innate immune genes, cytokine and chemokine genes, plasma membrane proteins, and CD markers (adhesion molecules), which may promote VSMCs plasticity and trans-differentiation not only to 7 reported cell types but also 21 new cell types. Transcriptomic remodeling and upregulation of nuclear membrane proteins, nucleoli proteins, and nucleoplasm proteins have been identified as the part of nuclear stress sensory system in aortic diseases and innate immune responses of VSMCs. In aortic diseases and VSMC innate immune responses, upregulated transcription factors are increasingly localized in nucleoplasm, and decreasingly localized in nucleoli and non-nuclear locations. The upregulations of innate immune genes, and other 12 lists of innate immune related genes are promoted by transcription factors AHR^–/–^, NF-kB^–/–^, ER stress promoting kinase PERK^–/–^, ROS enzyme NOX2^–/–^, trained immunity promoting histone lysine methyltransferase SET7^–/–^, but are more significantly suppressed by anti-oxidant transcription factor NRF2. Our new findings have provided novel insights on the roles of innate immunity in aortic diseases and VSMC pathologies and therapeutic targets for the development of new treatments for those diseases.

## Data availability statement

The original contributions presented in the study are included in the article/**Supplementary Material**. Further inquiries can be directed to the corresponding author.

## Author contributions

QY: Conceptualization, Formal analysis, Methodology, Software, Writing – original draft. FS: Conceptualization, Formal analysis, Investigation, Methodology, Software, Validation, Visualization, Writing – original draft. YL: Conceptualization, Writing – review & editing. YP: Formal analysis, Software, Writing – review & editing. KX: Conceptualization, Formal analysis, Writing – review & editing. YS: Conceptualization, Formal analysis, Writing – review & editing. XJ: Conceptualization, Formal analysis, Resources, Writing – review & editing. SW: Conceptualization, Writing – review & editing. LY: Conceptualization, Writing – review & editing. XL: Conceptualization, Writing – review & editing. AG: Conceptualization, Formal analysis, Writing – review & editing. JL: Conceptualization, Formal analysis, Writing – review & editing. XS: Formal analysis, Software, Writing – review & editing. HZ: Formal analysis, Software, Writing – review & editing. LM: Conceptualization, Writing – review & editing. RV-P: Conceptualization, Formal analysis, Writing – review & editing. YT: Conceptualization, Writing – review & editing. HW: Conceptualization, Formal analysis, Funding acquisition, Supervision, Writing – review & editing. XY: Conceptualization, Data curation, Formal analysis, Funding acquisition, Methodology, Project administration, Resources, Supervision, Visualization, Writing – original draft.
