# Supplementary material for: Innate immunity of vascular smooth muscle cells contributes to two-wave inflammation in atherosclerosis, twin-peak inflammation in aortic aneurysms and trans-differentiation potential into 25 cell types
Source: Front Immunol. 2024 Jan 24;14:1348238. doi: 10.3389/fimmu.2023.1348238 (PMC10847266; doi:10.3389/fimmu.2023.1348238)
Supplement: Supplementary file 1 [file Presentation_1.pptx]

## Slide 1
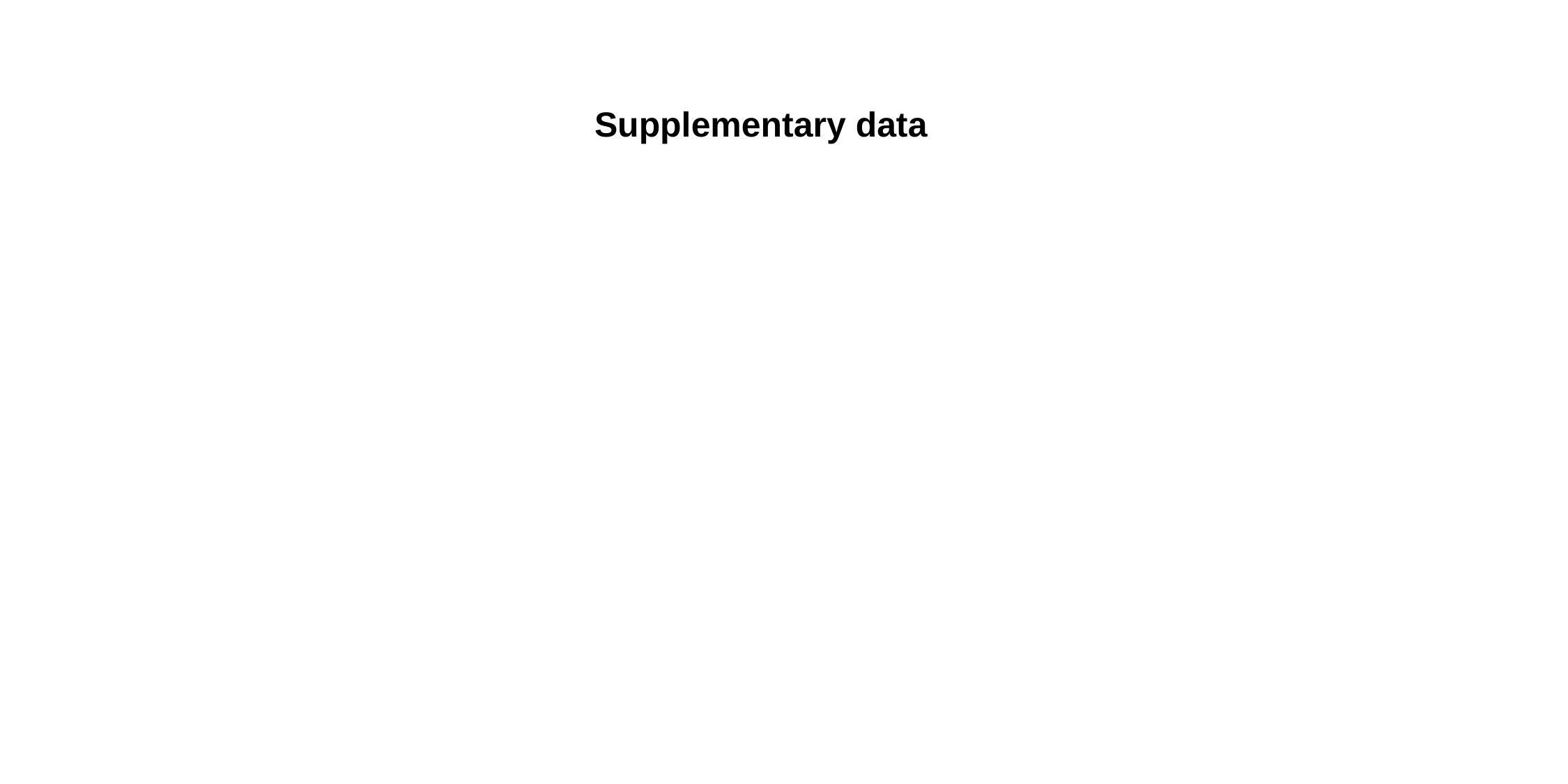

Supplementary data

## Slide 2
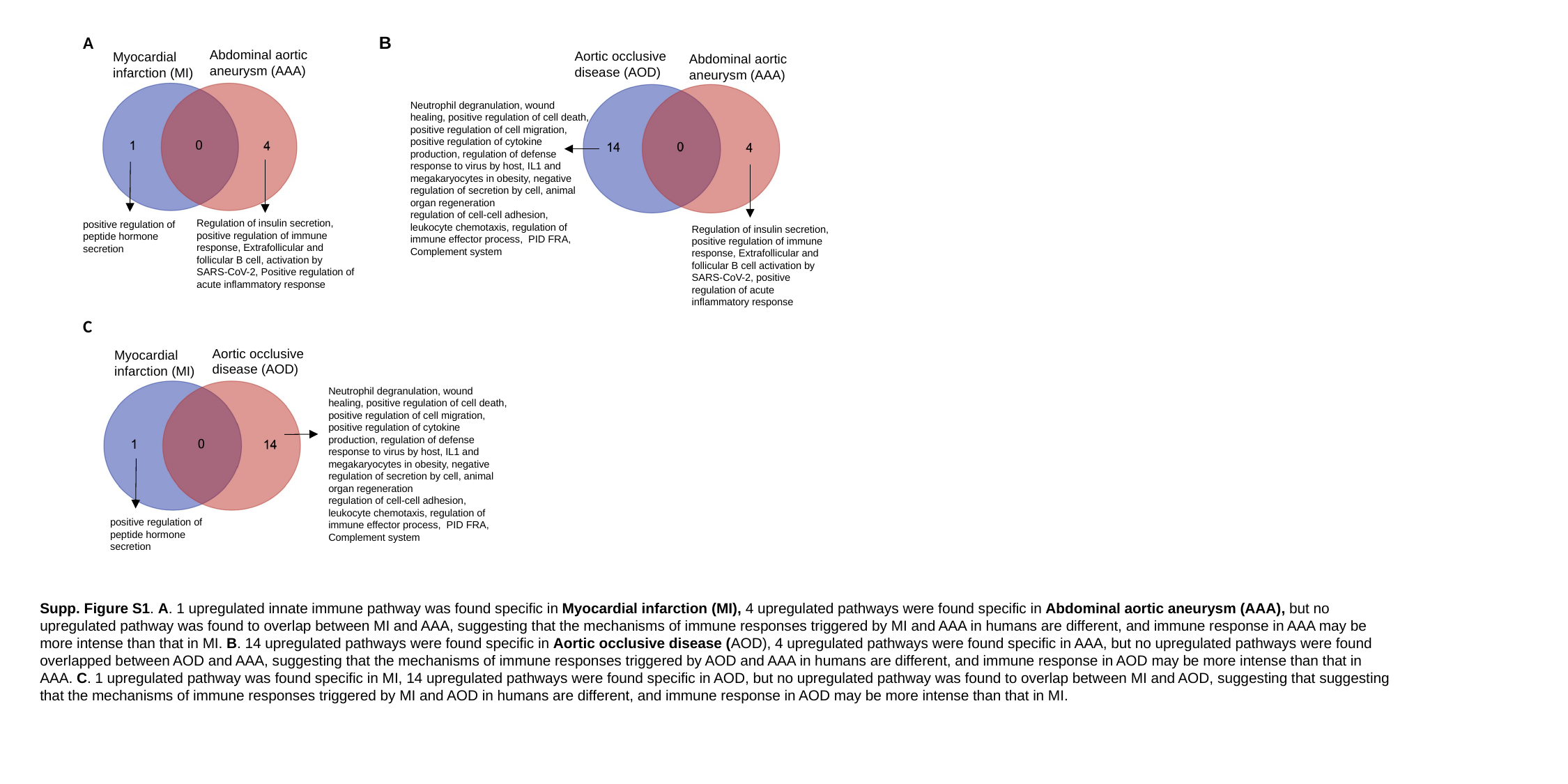

A
B
Aortic occlusive disease (AOD)
Abdominal aortic aneurysm (AAA)
Neutrophil degranulation, wound healing, positive regulation of cell death, positive regulation of cell migration, positive regulation of cytokine production, regulation of defense response to virus by host, IL1 and megakaryocytes in obesity, negative regulation of secretion by cell, animal organ regeneration
regulation of cell-cell adhesion, leukocyte chemotaxis, regulation of immune effector process, PID FRA, Complement system
Regulation of insulin secretion, positive regulation of immune response, Extrafollicular and follicular B cell activation by SARS-CoV-2, positive regulation of acute inflammatory response
Abdominal aortic aneurysm (AAA)
Myocardial infarction (MI)
Regulation of insulin secretion, positive regulation of immune response, Extrafollicular and follicular B cell, activation by SARS-CoV-2, Positive regulation of acute inflammatory response
positive regulation of peptide hormone secretion
C
Aortic occlusive disease (AOD)
Myocardial infarction (MI)
Neutrophil degranulation, wound healing, positive regulation of cell death, positive regulation of cell migration, positive regulation of cytokine production, regulation of defense response to virus by host, IL1 and megakaryocytes in obesity, negative regulation of secretion by cell, animal organ regeneration
regulation of cell-cell adhesion, leukocyte chemotaxis, regulation of immune effector process, PID FRA, Complement system
positive regulation of peptide hormone secretion
Supp. Figure S1. A. 1 upregulated innate immune pathway was found specific in Myocardial infarction (MI), 4 upregulated pathways were found specific in Abdominal aortic aneurysm (AAA), but no upregulated pathway was found to overlap between MI and AAA, suggesting that the mechanisms of immune responses triggered by MI and AAA in humans are different, and immune response in AAA may be more intense than that in MI. B. 14 upregulated pathways were found specific in Aortic occlusive disease (AOD), 4 upregulated pathways were found specific in AAA, but no upregulated pathways were found overlapped between AOD and AAA, suggesting that the mechanisms of immune responses triggered by AOD and AAA in humans are different, and immune response in AOD may be more intense than that in AAA. C. 1 upregulated pathway was found specific in MI, 14 upregulated pathways were found specific in AOD, but no upregulated pathway was found to overlap between MI and AOD, suggesting that suggesting that the mechanisms of immune responses triggered by MI and AOD in humans are different, and immune response in AOD may be more intense than that in MI.

## Slide 3
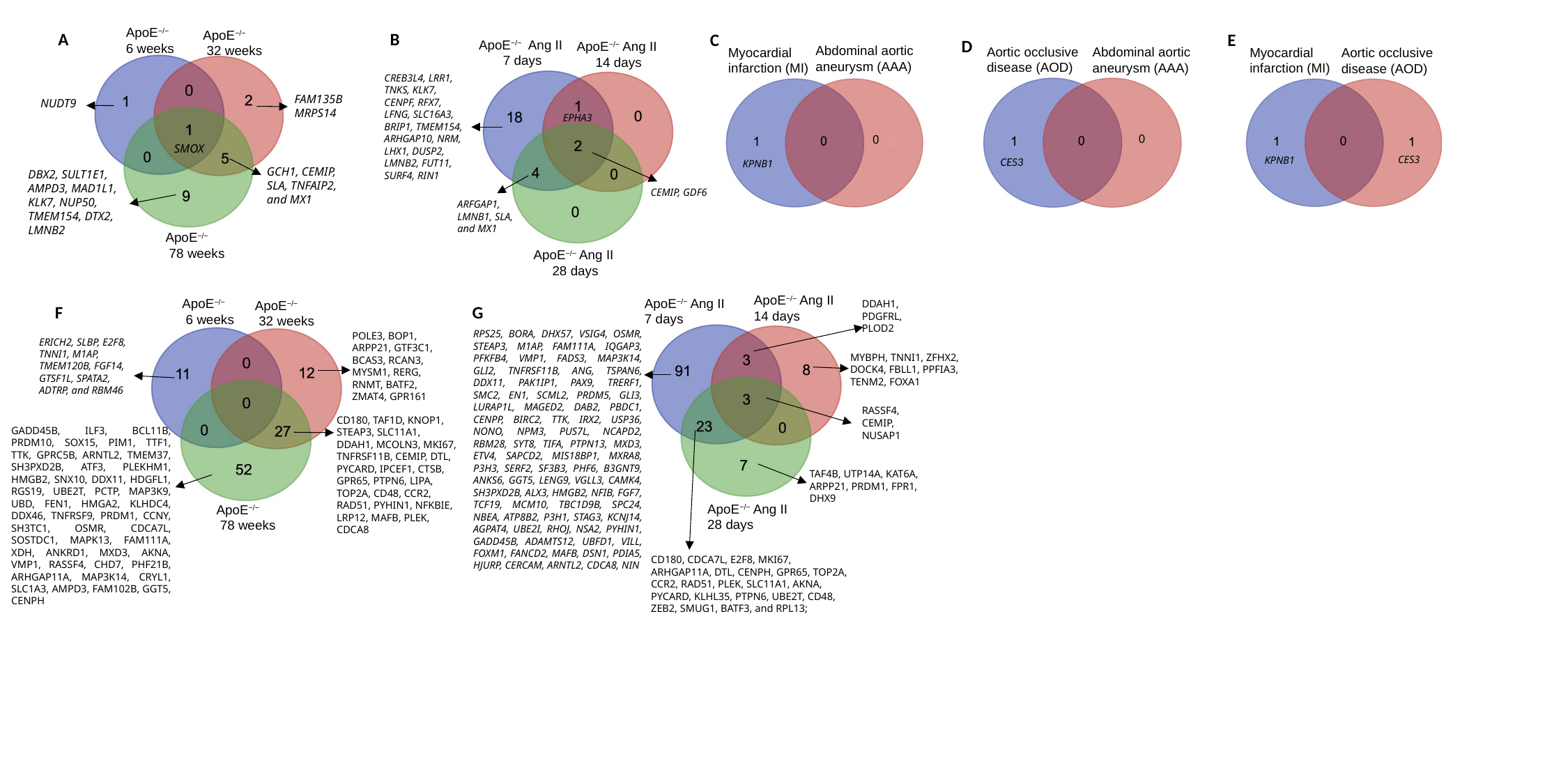

ApoE−/−
6 weeks
ApoE−/−
 32 weeks
ApoE−/−
 78 weeks
A
B
E
Myocardial infarction (MI)
Aortic occlusive disease (AOD)
CES3
KPNB1
C
D
Aortic occlusive disease (AOD)
Abdominal aortic aneurysm (AAA)
CES3
0
ApoE−/− Ang II
7 days
ApoE−/− Ang II
14 days
ApoE−/− Ang II
28 days
CREB3L4, LRR1, TNKS, KLK7, CENPF, RFX7, LFNG, SLC16A3, BRIP1, TMEM154, ARHGAP10, NRM, LHX1, DUSP2, LMNB2, FUT11, SURF4, RIN1
EPHA3
CEMIP, GDF6
ARFGAP1, LMNB1, SLA, and MX1
Abdominal aortic aneurysm (AAA)
Myocardial infarction (MI)
KPNB1
0
FAM135B
MRPS14
NUDT9
SMOX
GCH1, CEMIP, SLA, TNFAIP2, and MX1
DBX2, SULT1E1, AMPD3, MAD1L1, KLK7, NUP50, TMEM154, DTX2, LMNB2
ApoE−/− Ang II 14 days
ApoE−/− Ang II
7 days
ApoE−/− Ang II
28 days
DDAH1, PDGFRL, PLOD2
RPS25, BORA, DHX57, VSIG4, OSMR, STEAP3, M1AP, FAM111A, IQGAP3, PFKFB4, VMP1, FADS3, MAP3K14, GLI2, TNFRSF11B, ANG, TSPAN6, DDX11, PAK1IP1, PAX9, TRERF1, SMC2, EN1, SCML2, PRDM5, GLI3, LURAP1L, MAGED2, DAB2, PBDC1, CENPP, BIRC2, TTK, IRX2, USP36, NONO, NPM3, PUS7L, NCAPD2, RBM28, SYT8, TIFA, PTPN13, MXD3, ETV4, SAPCD2, MIS18BP1, MXRA8, P3H3, SERF2, SF3B3, PHF6, B3GNT9, ANKS6, GGT5, LENG9, VGLL3, CAMK4, SH3PXD2B, ALX3, HMGB2, NFIB, FGF7, TCF19, MCM10, TBC1D9B, SPC24, NBEA, ATP8B2, P3H1, STAG3, KCNJ14, AGPAT4, UBE2I, RHOJ, NSA2, PYHIN1, GADD45B, ADAMTS12, UBFD1, VILL, FOXM1, FANCD2, MAFB, DSN1, PDIA5, HJURP, CERCAM, ARNTL2, CDCA8, NIN
RASSF4, CEMIP, NUSAP1
TAF4B, UTP14A, KAT6A, ARPP21, PRDM1, FPR1, DHX9
CD180, CDCA7L, E2F8, MKI67, ARHGAP11A, DTL, CENPH, GPR65, TOP2A, CCR2, RAD51, PLEK, SLC11A1, AKNA, PYCARD, KLHL35, PTPN6, UBE2T, CD48, ZEB2, SMUG1, BATF3, and RPL13;
MYBPH, TNNI1, ZFHX2, DOCK4, FBLL1, PPFIA3, TENM2, FOXA1
ApoE−/−
 6 weeks
ApoE−/−
 32 weeks
ApoE−/−
 78 weeks
F
POLE3, BOP1, ARPP21, GTF3C1, BCAS3, RCAN3, MYSM1, RERG, RNMT, BATF2, ZMAT4, GPR161
ERICH2, SLBP, E2F8, TNNI1, M1AP, TMEM120B, FGF14, GTSF1L, SPATA2, ADTRP, and RBM46
CD180, TAF1D, KNOP1, STEAP3, SLC11A1, DDAH1, MCOLN3, MKI67, TNFRSF11B, CEMIP, DTL, PYCARD, IPCEF1, CTSB, GPR65, PTPN6, LIPA, TOP2A, CD48, CCR2, RAD51, PYHIN1, NFKBIE, LRP12, MAFB, PLEK, CDCA8
GADD45B, ILF3, BCL11B, PRDM10, SOX15, PIM1, TTF1, TTK, GPRC5B, ARNTL2, TMEM37, SH3PXD2B, ATF3, PLEKHM1, HMGB2, SNX10, DDX11, HDGFL1, RGS19, UBE2T, PCTP, MAP3K9, UBD, FEN1, HMGA2, KLHDC4, DDX46, TNFRSF9, PRDM1, CCNY, SH3TC1, OSMR, CDCA7L, SOSTDC1, MAPK13, FAM111A, XDH, ANKRD1, MXD3, AKNA, VMP1, RASSF4, CHD7, PHF21B, ARHGAP11A, MAP3K14, CRYL1, SLC1A3, AMPD3, FAM102B, GGT5, CENPH
G

## Slide 4
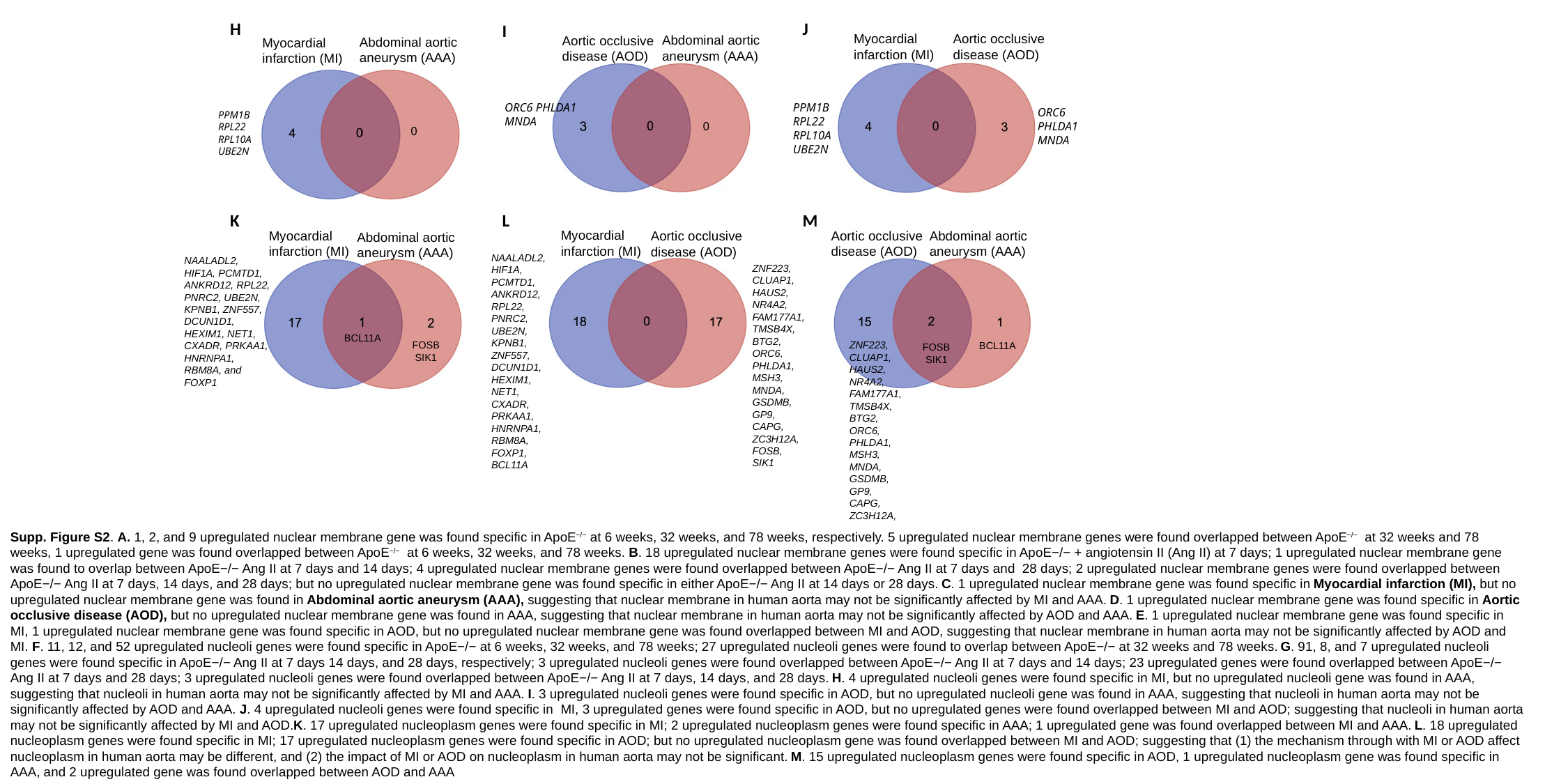

I
Abdominal aortic aneurysm (AAA)
Aortic occlusive disease (AOD)
ORC6 PHLDA1 MNDA
0
Myocardial infarction (MI)
PPM1B RPL22 RPL10A UBE2N
0
Abdominal aortic aneurysm (AAA)
H
J
Myocardial infarction (MI)
Aortic occlusive disease (AOD)
PPM1B
RPL22 RPL10A UBE2N
ORC6 PHLDA1
MNDA
K
M
L
Myocardial infarction (MI)
Aortic occlusive disease (AOD)
Aortic occlusive disease (AOD)
Abdominal aortic aneurysm (AAA)
Myocardial infarction (MI)
Abdominal aortic aneurysm (AAA)
NAALADL2, HIF1A, PCMTD1, ANKRD12, RPL22, PNRC2, UBE2N, KPNB1, ZNF557, DCUN1D1, HEXIM1, NET1, CXADR, PRKAA1, HNRNPA1, RBM8A, FOXP1, BCL11A
NAALADL2, HIF1A, PCMTD1, ANKRD12, RPL22, PNRC2, UBE2N, KPNB1, ZNF557, DCUN1D1, HEXIM1, NET1, CXADR, PRKAA1, HNRNPA1, RBM8A, and FOXP1
ZNF223, CLUAP1, HAUS2, NR4A2, FAM177A1, TMSB4X, BTG2, ORC6, PHLDA1, MSH3, MNDA, GSDMB, GP9, CAPG, ZC3H12A, FOSB,
SIK1
BCL11A
FOSB
 SIK1
ZNF223, CLUAP1, HAUS2, NR4A2, FAM177A1, TMSB4X, BTG2, ORC6, PHLDA1, MSH3, MNDA, GSDMB, GP9, CAPG, ZC3H12A,
BCL11A
FOSB
 SIK1
Supp. Figure S2. A. 1, 2, and 9 upregulated nuclear membrane gene was found specific in ApoE−/− at 6 weeks, 32 weeks, and 78 weeks, respectively. 5 upregulated nuclear membrane genes were found overlapped between ApoE−/− at 32 weeks and 78 weeks, 1 upregulated gene was found overlapped between ApoE−/− at 6 weeks, 32 weeks, and 78 weeks. B. 18 upregulated nuclear membrane genes were found specific in ApoE−/− + angiotensin II (Ang II) at 7 days; 1 upregulated nuclear membrane gene was found to overlap between ApoE−/− Ang II at 7 days and 14 days; 4 upregulated nuclear membrane genes were found overlapped between ApoE−/− Ang II at 7 days and 28 days; 2 upregulated nuclear membrane genes were found overlapped between ApoE−/− Ang II at 7 days, 14 days, and 28 days; but no upregulated nuclear membrane gene was found specific in either ApoE−/− Ang II at 14 days or 28 days. C. 1 upregulated nuclear membrane gene was found specific in Myocardial infarction (MI), but no upregulated nuclear membrane gene was found in Abdominal aortic aneurysm (AAA), suggesting that nuclear membrane in human aorta may not be significantly affected by MI and AAA. D. 1 upregulated nuclear membrane gene was found specific in Aortic occlusive disease (AOD), but no upregulated nuclear membrane gene was found in AAA, suggesting that nuclear membrane in human aorta may not be significantly affected by AOD and AAA. E. 1 upregulated nuclear membrane gene was found specific in MI, 1 upregulated nuclear membrane gene was found specific in AOD, but no upregulated nuclear membrane gene was found overlapped between MI and AOD, suggesting that nuclear membrane in human aorta may not be significantly affected by AOD and MI. F. 11, 12, and 52 upregulated nucleoli genes were found specific in ApoE−/− at 6 weeks, 32 weeks, and 78 weeks; 27 upregulated nucleoli genes were found to overlap between ApoE−/− at 32 weeks and 78 weeks. G. 91, 8, and 7 upregulated nucleoli genes were found specific in ApoE−/− Ang II at 7 days 14 days, and 28 days, respectively; 3 upregulated nucleoli genes were found overlapped between ApoE−/− Ang II at 7 days and 14 days; 23 upregulated genes were found overlapped between ApoE−/− Ang II at 7 days and 28 days; 3 upregulated nucleoli genes were found overlapped between ApoE−/− Ang II at 7 days, 14 days, and 28 days. H. 4 upregulated nucleoli genes were found specific in MI, but no upregulated nucleoli gene was found in AAA, suggesting that nucleoli in human aorta may not be significantly affected by MI and AAA. I. 3 upregulated nucleoli genes were found specific in AOD, but no upregulated nucleoli gene was found in AAA, suggesting that nucleoli in human aorta may not be significantly affected by AOD and AAA. J. 4 upregulated nucleoli genes were found specific in MI, 3 upregulated genes were found specific in AOD, but no upregulated genes were found overlapped between MI and AOD; suggesting that nucleoli in human aorta may not be significantly affected by MI and AOD.K. 17 upregulated nucleoplasm genes were found specific in MI; 2 upregulated nucleoplasm genes were found specific in AAA; 1 upregulated gene was found overlapped between MI and AAA. L. 18 upregulated nucleoplasm genes were found specific in MI; 17 upregulated nucleoplasm genes were found specific in AOD; but no upregulated nucleoplasm gene was found overlapped between MI and AOD; suggesting that (1) the mechanism through with MI or AOD affect nucleoplasm in human aorta may be different, and (2) the impact of MI or AOD on nucleoplasm in human aorta may not be significant. M. 15 upregulated nucleoplasm genes were found specific in AOD, 1 upregulated nucleoplasm gene was found specific in AAA, and 2 upregulated gene was found overlapped between AOD and AAA

## Slide 5
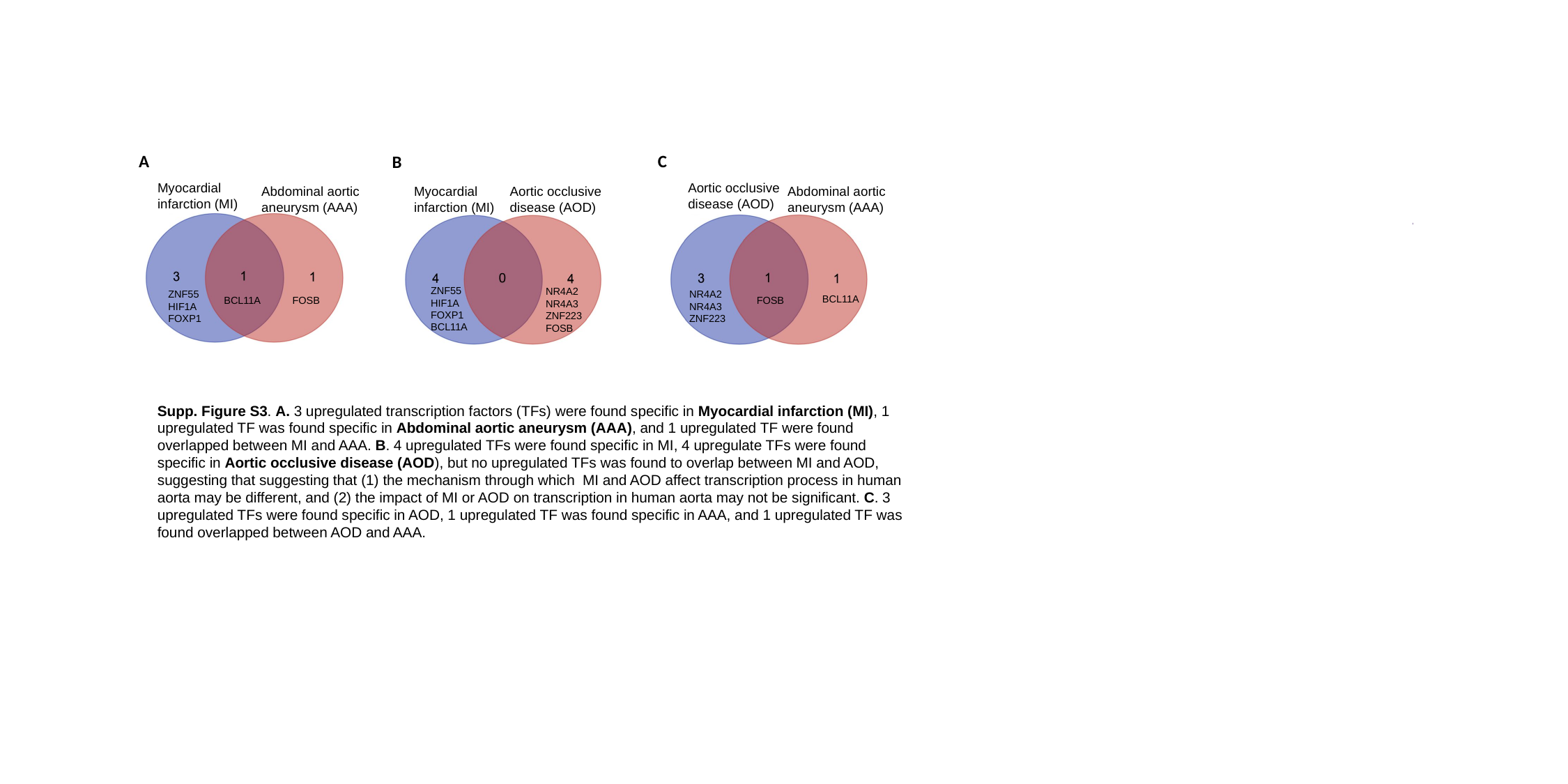

A
C
B
Myocardial infarction (MI)
Abdominal aortic aneurysm (AAA)
Aortic occlusive disease (AOD)
Abdominal aortic aneurysm (AAA)
Myocardial infarction (MI)
Aortic occlusive disease (AOD)
ZNF55
HIF1A
FOXP1
BCL11A
NR4A2
NR4A3
ZNF223
FOSB
ZNF55
HIF1A
FOXP1
NR4A2
NR4A3
ZNF223
BCL11A
BCL11A
FOSB
FOSB
Supp. Figure S3. A. 3 upregulated transcription factors (TFs) were found specific in Myocardial infarction (MI), 1 upregulated TF was found specific in Abdominal aortic aneurysm (AAA), and 1 upregulated TF were found overlapped between MI and AAA. B. 4 upregulated TFs were found specific in MI, 4 upregulate TFs were found specific in Aortic occlusive disease (AOD), but no upregulated TFs was found to overlap between MI and AOD, suggesting that suggesting that (1) the mechanism through which MI and AOD affect transcription process in human aorta may be different, and (2) the impact of MI or AOD on transcription in human aorta may not be significant. C. 3 upregulated TFs were found specific in AOD, 1 upregulated TF was found specific in AAA, and 1 upregulated TF was found overlapped between AOD and AAA.

## Slide 6
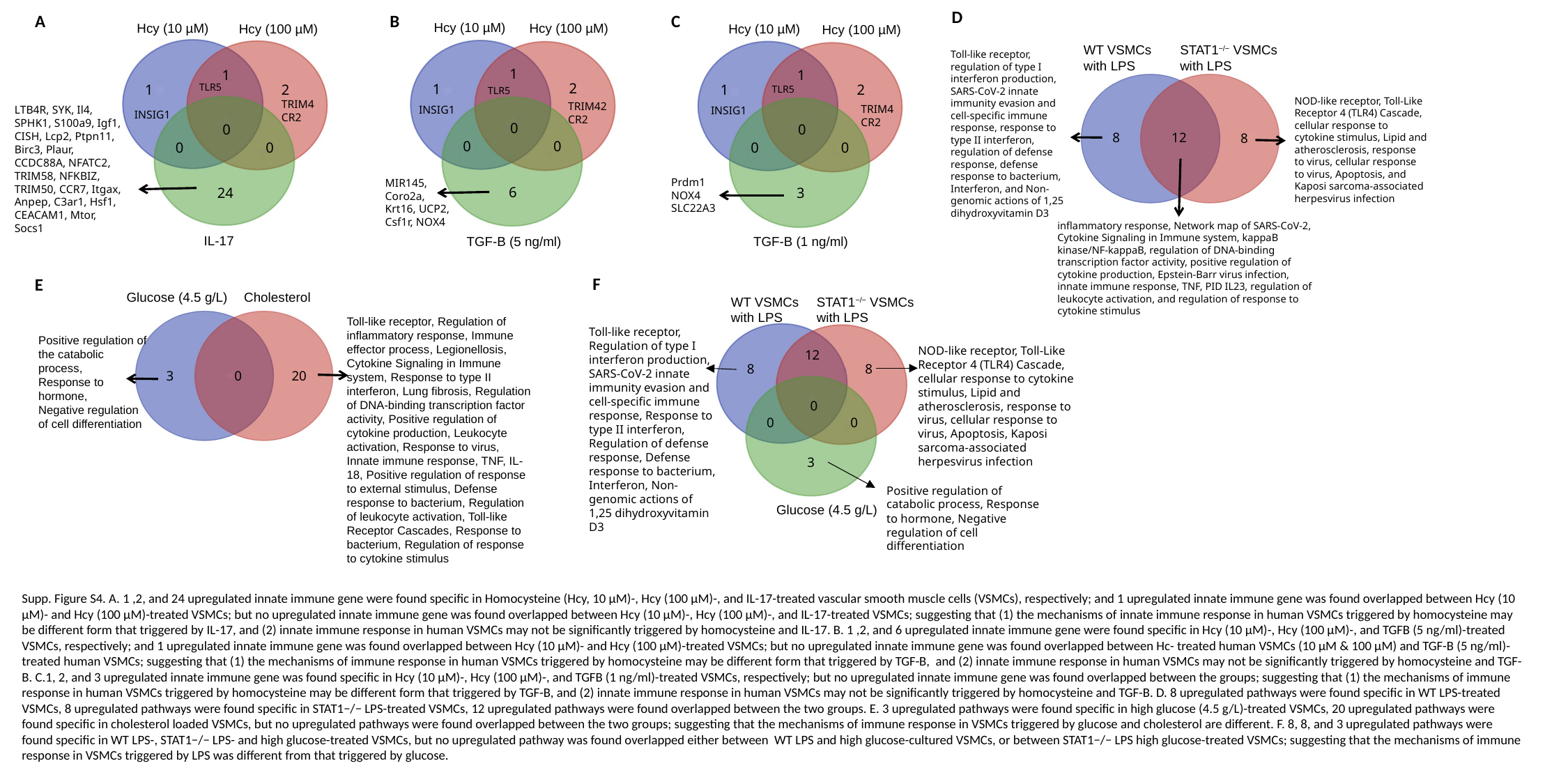

D
STAT1−/− VSMCs with LPS
WT VSMCs with LPS
Toll-like receptor, regulation of type I interferon production, SARS-CoV-2 innate immunity evasion and cell-specific immune response, response to type II interferon, regulation of defense response, defense response to bacterium, Interferon, and Non-genomic actions of 1,25 dihydroxyvitamin D3
NOD-like receptor, Toll-Like Receptor 4 (TLR4) Cascade, cellular response to cytokine stimulus, Lipid and atherosclerosis, response to virus, cellular response to virus, Apoptosis, and Kaposi sarcoma-associated herpesvirus infection
inflammatory response, Network map of SARS-CoV-2, Cytokine Signaling in Immune system, kappaB kinase/NF-kappaB, regulation of DNA-binding transcription factor activity, positive regulation of cytokine production, Epstein-Barr virus infection, innate immune response, TNF, PID IL23, regulation of leukocyte activation, and regulation of response to cytokine stimulus
8
12
8
A
C
1
2
1
TLR5
TRIM4
CR2
INSIG1
0
0
0
Prdm1
NOX4 SLC22A3
3
TGF-B (1 ng/ml)
B
Hcy (10 µM)
Hcy (100 µM)
1
2
1
TLR5
TRIM42
CR2
INSIG1
0
0
0
MIR145, Coro2a, Krt16, UCP2, Csf1r, NOX4
6
TGF-B (5 ng/ml)
Hcy (10 µM)
1
TLR5
2
1
TRIM4
CR2
LTB4R, SYK, Il4, SPHK1, S100a9, Igf1, CISH, Lcp2, Ptpn11, Birc3, Plaur, CCDC88A, NFATC2, TRIM58, NFKBIZ, TRIM50, CCR7, Itgax, Anpep, C3ar1, Hsf1, CEACAM1, Mtor, Socs1
INSIG1
0
0
0
24
IL-17
Hcy (100 µM)
Hcy (10 µM)
Hcy (100 µM)
F
WT VSMCs with LPS
STAT1−/− VSMCs with LPS
Toll-like receptor, Regulation of type I interferon production, SARS-CoV-2 innate immunity evasion and cell-specific immune response, Response to type II interferon, Regulation of defense response, Defense response to bacterium, Interferon, Non-genomic actions of 1,25 dihydroxyvitamin D3
NOD-like receptor, Toll-Like Receptor 4 (TLR4) Cascade, cellular response to cytokine stimulus, Lipid and atherosclerosis, response to virus, cellular response to virus, Apoptosis, Kaposi sarcoma-associated herpesvirus infection
12
8
8
0
0
0
3
Positive regulation of catabolic process, Response to hormone, Negative regulation of cell differentiation
Glucose (4.5 g/L)
E
Glucose (4.5 g/L)
Cholesterol
3
0
20
Toll-like receptor, Regulation of inflammatory response, Immune effector process, Legionellosis, Cytokine Signaling in Immune system, Response to type II interferon, Lung fibrosis, Regulation of DNA-binding transcription factor activity, Positive regulation of cytokine production, Leukocyte activation, Response to virus, Innate immune response, TNF, IL-18, Positive regulation of response to external stimulus, Defense response to bacterium, Regulation of leukocyte activation, Toll-like Receptor Cascades, Response to bacterium, Regulation of response to cytokine stimulus
Positive regulation of the catabolic process,
Response to hormone,
Negative regulation of cell differentiation
Supp. Figure S4. A. 1 ,2, and 24 upregulated innate immune gene were found specific in Homocysteine (Hcy, 10 µM)-, Hcy (100 µM)-, and IL-17-treated vascular smooth muscle cells (VSMCs), respectively; and 1 upregulated innate immune gene was found overlapped between Hcy (10 µM)- and Hcy (100 µM)-treated VSMCs; but no upregulated innate immune gene was found overlapped between Hcy (10 µM)-, Hcy (100 µM)-, and IL-17-treated VSMCs; suggesting that (1) the mechanisms of innate immune response in human VSMCs triggered by homocysteine may be different form that triggered by IL-17, and (2) innate immune response in human VSMCs may not be significantly triggered by homocysteine and IL-17. B. 1 ,2, and 6 upregulated innate immune gene were found specific in Hcy (10 µM)-, Hcy (100 µM)-, and TGFB (5 ng/ml)-treated VSMCs, respectively; and 1 upregulated innate immune gene was found overlapped between Hcy (10 µM)- and Hcy (100 µM)-treated VSMCs; but no upregulated innate immune gene was found overlapped between Hc- treated human VSMCs (10 µM & 100 µM) and TGF-B (5 ng/ml)-treated human VSMCs; suggesting that (1) the mechanisms of immune response in human VSMCs triggered by homocysteine may be different form that triggered by TGF-B, and (2) innate immune response in human VSMCs may not be significantly triggered by homocysteine and TGF-B. C.1, 2, and 3 upregulated innate immune gene was found specific in Hcy (10 µM)-, Hcy (100 µM)-, and TGFB (1 ng/ml)-treated VSMCs, respectively; but no upregulated innate immune gene was found overlapped between the groups; suggesting that (1) the mechanisms of immune response in human VSMCs triggered by homocysteine may be different form that triggered by TGF-B, and (2) innate immune response in human VSMCs may not be significantly triggered by homocysteine and TGF-B. D. 8 upregulated pathways were found specific in WT LPS-treated VSMCs, 8 upregulated pathways were found specific in STAT1−/− LPS-treated VSMCs, 12 upregulated pathways were found overlapped between the two groups. E. 3 upregulated pathways were found specific in high glucose (4.5 g/L)-treated VSMCs, 20 upregulated pathways were found specific in cholesterol loaded VSMCs, but no upregulated pathways were found overlapped between the two groups; suggesting that the mechanisms of immune response in VSMCs triggered by glucose and cholesterol are different. F. 8, 8, and 3 upregulated pathways were found specific in WT LPS-, STAT1−/− LPS- and high glucose-treated VSMCs, but no upregulated pathway was found overlapped either between WT LPS and high glucose-cultured VSMCs, or between STAT1−/− LPS high glucose-treated VSMCs; suggesting that the mechanisms of immune response in VSMCs triggered by LPS was different from that triggered by glucose.

## Slide 7
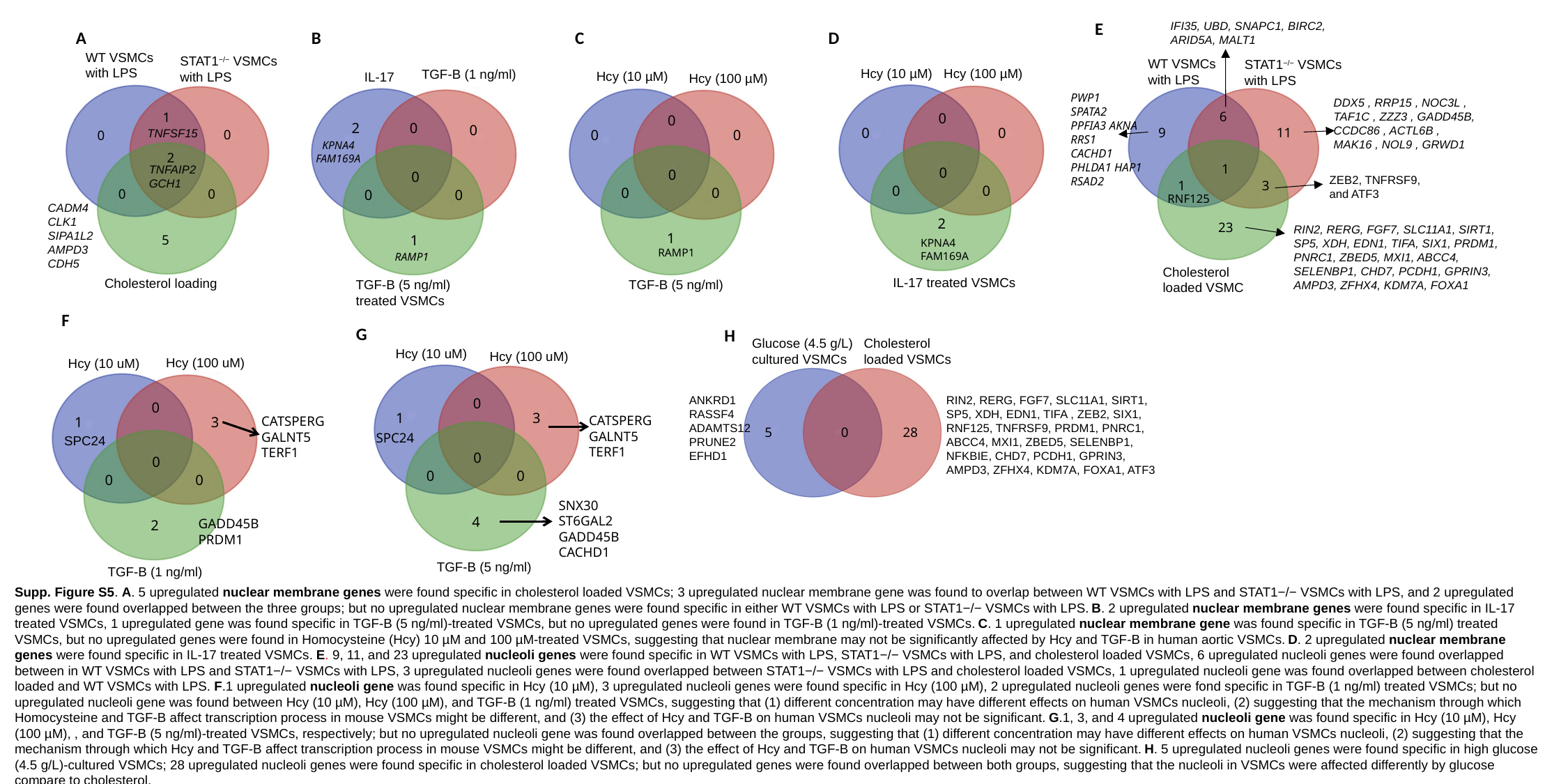

E
IFI35, UBD, SNAPC1, BIRC2, ARID5A, MALT1
WT VSMCs with LPS
STAT1−/− VSMCs with LPS
6
11
9
1
1
3
23
Cholesterol loaded VSMC
PWP1
SPATA2 PPFIA3 AKNA
RRS1
CACHD1 PHLDA1 HAP1 RSAD2
DDX5 , RRP15 , NOC3L , TAF1C , ZZZ3 , GADD45B, CCDC86 , ACTL6B , MAK16 , NOL9 , GRWD1
ZEB2, TNFRSF9, and ATF3
RNF125
RIN2, RERG, FGF7, SLC11A1, SIRT1, SP5, XDH, EDN1, TIFA, SIX1, PRDM1, PNRC1, ZBED5, MXI1, ABCC4, SELENBP1, CHD7, PCDH1, GPRIN3, AMPD3, ZFHX4, KDM7A, FOXA1
D
A
B
C
WT VSMCs with LPS
STAT1−/− VSMCs with LPS
1
0
0
2
0
0
5
Cholesterol loading
Hcy (100 µM)
Hcy (10 µM)
TGF-B (1 ng/ml)
IL-17
2
0
0
KPNA4
FAM169A
0
0
0
1
RAMP1
TGF-B (5 ng/ml) treated VSMCs
Hcy (10 µM)
Hcy (100 µM)
0
0
0
0
0
0
1
RAMP1
TGF-B (5 ng/ml)
0
0
0
0
0
0
2
KPNA4
FAM169A
IL-17 treated VSMCs
TNFSF15
TNFAIP2
GCH1
CADM4
CLK1
SIPA1L2
AMPD3
CDH5
F
G
H
Glucose (4.5 g/L)
cultured VSMCs
Cholesterol loaded VSMCs
5
28
0
Hcy (10 uM)
Hcy (100 uM)
0
3
1
SPC24
0
0
0
SNX30
ST6GAL2
GADD45B
CACHD1
4
TGF-B (5 ng/ml)
CATSPERG
GALNT5
TERF1
Hcy (100 uM)
Hcy (10 uM)
0
CATSPERG
GALNT5
TERF1
3
1
SPC24
0
0
0
GADD45B
PRDM1
2
TGF-B (1 ng/ml)
ANKRD1
RASSF4
ADAMTS12
PRUNE2
EFHD1
RIN2, RERG, FGF7, SLC11A1, SIRT1, SP5, XDH, EDN1, TIFA , ZEB2, SIX1, RNF125, TNFRSF9, PRDM1, PNRC1, ABCC4, MXI1, ZBED5, SELENBP1, NFKBIE, CHD7, PCDH1, GPRIN3, AMPD3, ZFHX4, KDM7A, FOXA1, ATF3
Supp. Figure S5. A. 5 upregulated nuclear membrane genes were found specific in cholesterol loaded VSMCs; 3 upregulated nuclear membrane gene was found to overlap between WT VSMCs with LPS and STAT1−/− VSMCs with LPS, and 2 upregulated genes were found overlapped between the three groups; but no upregulated nuclear membrane genes were found specific in either WT VSMCs with LPS or STAT1−/− VSMCs with LPS. B. 2 upregulated nuclear membrane genes were found specific in IL-17 treated VSMCs, 1 upregulated gene was found specific in TGF-B (5 ng/ml)-treated VSMCs, but no upregulated genes were found in TGF-B (1 ng/ml)-treated VSMCs. C. 1 upregulated nuclear membrane gene was found specific in TGF-B (5 ng/ml) treated VSMCs, but no upregulated genes were found in Homocysteine (Hcy) 10 µM and 100 µM-treated VSMCs, suggesting that nuclear membrane may not be significantly affected by Hcy and TGF-B in human aortic VSMCs. D. 2 upregulated nuclear membrane genes were found specific in IL-17 treated VSMCs. E. 9, 11, and 23 upregulated nucleoli genes were found specific in WT VSMCs with LPS, STAT1−/− VSMCs with LPS, and cholesterol loaded VSMCs, 6 upregulated nucleoli genes were found overlapped between in WT VSMCs with LPS and STAT1−/− VSMCs with LPS, 3 upregulated nucleoli genes were found overlapped between STAT1−/− VSMCs with LPS and cholesterol loaded VSMCs, 1 upregulated nucleoli gene was found overlapped between cholesterol loaded and WT VSMCs with LPS. F.1 upregulated nucleoli gene was found specific in Hcy (10 µM), 3 upregulated nucleoli genes were found specific in Hcy (100 µM), 2 upregulated nucleoli genes were fond specific in TGF-B (1 ng/ml) treated VSMCs; but no upregulated nucleoli gene was found between Hcy (10 µM), Hcy (100 µM), and TGF-B (1 ng/ml) treated VSMCs, suggesting that (1) different concentration may have different effects on human VSMCs nucleoli, (2) suggesting that the mechanism through which Homocysteine and TGF-B affect transcription process in mouse VSMCs might be different, and (3) the effect of Hcy and TGF-B on human VSMCs nucleoli may not be significant. G.1, 3, and 4 upregulated nucleoli gene was found specific in Hcy (10 µM), Hcy (100 µM), , and TGF-B (5 ng/ml)-treated VSMCs, respectively; but no upregulated nucleoli gene was found overlapped between the groups, suggesting that (1) different concentration may have different effects on human VSMCs nucleoli, (2) suggesting that the mechanism through which Hcy and TGF-B affect transcription process in mouse VSMCs might be different, and (3) the effect of Hcy and TGF-B on human VSMCs nucleoli may not be significant. H. 5 upregulated nucleoli genes were found specific in high glucose (4.5 g/L)-cultured VSMCs; 28 upregulated nucleoli genes were found specific in cholesterol loaded VSMCs; but no upregulated genes were found overlapped between both groups, suggesting that the nucleoli in VSMCs were affected differently by glucose compare to cholesterol.

## Slide 8
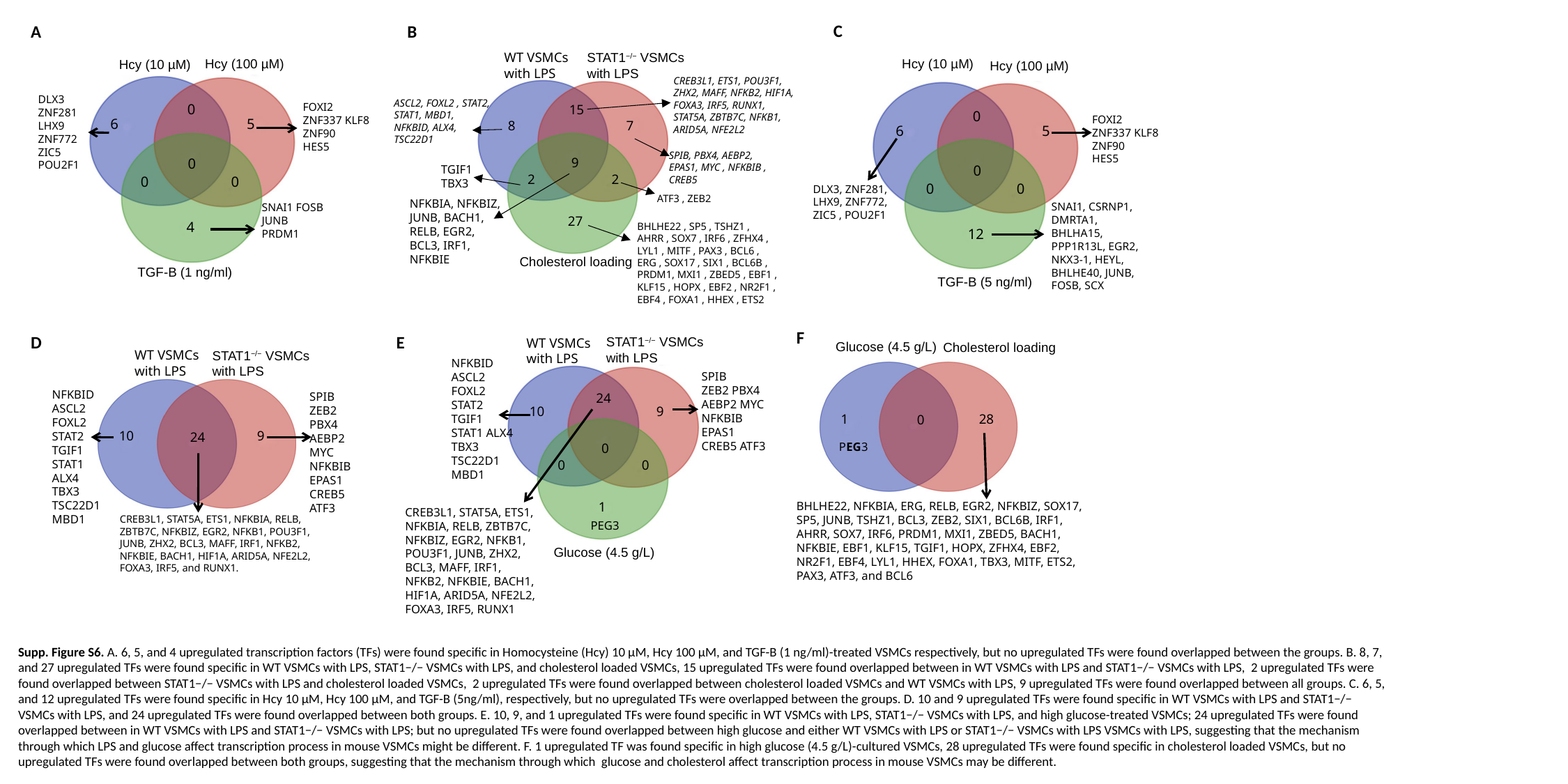

C
A
B
WT VSMCs with LPS
15
7
8
9
2
2
27
Cholesterol loading
CREB3L1, ETS1, POU3F1, ZHX2, MAFF, NFKB2, HIF1A, FOXA3, IRF5, RUNX1, STAT5A, ZBTB7C, NFKB1, ARID5A, NFE2L2
ASCL2, FOXL2 , STAT2, STAT1, MBD1, NFKBID, ALX4, TSC22D1
SPIB, PBX4, AEBP2, EPAS1, MYC , NFKBIB , CREB5
TGIF1
TBX3
ATF3 , ZEB2
NFKBIA, NFKBIZ, JUNB, BACH1, RELB, EGR2, BCL3, IRF1, NFKBIE
BHLHE22 , SP5 , TSHZ1 , AHRR , SOX7 , IRF6 , ZFHX4 , LYL1 , MITF , PAX3 , BCL6 , ERG , SOX17 , SIX1 , BCL6B , PRDM1, MXI1 , ZBED5 , EBF1 , KLF15 , HOPX , EBF2 , NR2F1 , EBF4 , FOXA1 , HHEX , ETS2
STAT1−/− VSMCs with LPS
Hcy (10 µM)
Hcy (100 µM)
Hcy (10 µM)
DLX3 ZNF281 LHX9 ZNF772
ZIC5
POU2F1
0
FOXI2 ZNF337 KLF8
ZNF90
HES5
5
6
0
0
0
SNAI1 FOSB
JUNB PRDM1
4
TGF-B (1 ng/ml)
Hcy (100 µM)
0
FOXI2 ZNF337 KLF8
ZNF90
HES5
6
5
0
0
0
DLX3, ZNF281, LHX9, ZNF772, ZIC5 , POU2F1
SNAI1, CSRNP1, DMRTA1, BHLHA15, PPP1R13L, EGR2, NKX3-1, HEYL, BHLHE40, JUNB, FOSB, SCX
12
TGF-B (5 ng/ml)
F
D
E
STAT1−/− VSMCs with LPS
WT VSMCs with LPS
NFKBID ASCL2 FOXL2 STAT2 TGIF1 STAT1 ALX4
TBX3 TSC22D1 MBD1
SPIB
ZEB2 PBX4
AEBP2 MYC NFKBIB EPAS1 CREB5 ATF3
24
9
10
0
0
0
1
CREB3L1, STAT5A, ETS1, NFKBIA, RELB, ZBTB7C, NFKBIZ, EGR2, NFKB1, POU3F1, JUNB, ZHX2, BCL3, MAFF, IRF1, NFKB2, NFKBIE, BACH1, HIF1A, ARID5A, NFE2L2, FOXA3, IRF5, RUNX1
PEG3
Glucose (4.5 g/L)
Glucose (4.5 g/L)
Cholesterol loading
1
28
0
PEG3
BHLHE22, NFKBIA, ERG, RELB, EGR2, NFKBIZ, SOX17, SP5, JUNB, TSHZ1, BCL3, ZEB2, SIX1, BCL6B, IRF1, AHRR, SOX7, IRF6, PRDM1, MXI1, ZBED5, BACH1, NFKBIE, EBF1, KLF15, TGIF1, HOPX, ZFHX4, EBF2, NR2F1, EBF4, LYL1, HHEX, FOXA1, TBX3, MITF, ETS2, PAX3, ATF3, and BCL6
WT VSMCs with LPS
STAT1−/− VSMCs with LPS
10
9
24
NFKBID
ASCL2
FOXL2
STAT2
TGIF1
STAT1
ALX4
TBX3
TSC22D1
MBD1
SPIB
ZEB2
PBX4
AEBP2
MYC
NFKBIB
EPAS1
CREB5
ATF3
CREB3L1, STAT5A, ETS1, NFKBIA, RELB, ZBTB7C, NFKBIZ, EGR2, NFKB1, POU3F1, JUNB, ZHX2, BCL3, MAFF, IRF1, NFKB2, NFKBIE, BACH1, HIF1A, ARID5A, NFE2L2, FOXA3, IRF5, and RUNX1.
Supp. Figure S6. A. 6, 5, and 4 upregulated transcription factors (TFs) were found specific in Homocysteine (Hcy) 10 µM, Hcy 100 µM, and TGF-B (1 ng/ml)-treated VSMCs respectively, but no upregulated TFs were found overlapped between the groups. B. 8, 7, and 27 upregulated TFs were found specific in WT VSMCs with LPS, STAT1−/− VSMCs with LPS, and cholesterol loaded VSMCs, 15 upregulated TFs were found overlapped between in WT VSMCs with LPS and STAT1−/− VSMCs with LPS, 2 upregulated TFs were found overlapped between STAT1−/− VSMCs with LPS and cholesterol loaded VSMCs, 2 upregulated TFs were found overlapped between cholesterol loaded VSMCs and WT VSMCs with LPS, 9 upregulated TFs were found overlapped between all groups. C. 6, 5, and 12 upregulated TFs were found specific in Hcy 10 µM, Hcy 100 µM, and TGF-B (5ng/ml), respectively, but no upregulated TFs were overlapped between the groups. D. 10 and 9 upregulated TFs were found specific in WT VSMCs with LPS and STAT1−/− VSMCs with LPS, and 24 upregulated TFs were found overlapped between both groups. E. 10, 9, and 1 upregulated TFs were found specific in WT VSMCs with LPS, STAT1−/− VSMCs with LPS, and high glucose-treated VSMCs; 24 upregulated TFs were found overlapped between in WT VSMCs with LPS and STAT1−/− VSMCs with LPS; but no upregulated TFs were found overlapped between high glucose and either WT VSMCs with LPS or STAT1−/− VSMCs with LPS VSMCs with LPS, suggesting that the mechanism through which LPS and glucose affect transcription process in mouse VSMCs might be different. F. 1 upregulated TF was found specific in high glucose (4.5 g/L)-cultured VSMCs, 28 upregulated TFs were found specific in cholesterol loaded VSMCs, but no upregulated TFs were found overlapped between both groups, suggesting that the mechanism through which glucose and cholesterol affect transcription process in mouse VSMCs may be different.
